# Absolute Steady-State Thermal Conductivity Measurements by Use of a Transient Hot-Wire System

**DOI:** 10.6028/jres.105.028

**Published:** 2000-04-01

**Authors:** Hans M. Roder, Richard A. Perkins, Arno Laesecke, Carlos A. Nieto de Castro

**Affiliations:** National Institute of Standards and Technology, Boulder, CO 80303, USA; Departamento de Qumica and Centro de Cincias da Universidade de Lisboa, 1700 Lisboa, Portugal

**Keywords:** argon, convection, dilute gas, hot wire instrument, steady state, thermal conductivity, transient

## Abstract

A transient hot-wire apparatus was used to measure the thermal conductivity of argon with both steady-state and transient methods. The effects of wire diameter, eccentricity of the wire in the cavity, axial conduction, and natural convection were accounted for in the analysis of the steady-state measurements. Based on measurements on argon, the relative uncertainty at the 95 % level of confidence of the new steady-state measurements is 2 % at low densities. Using the same hot wires, the relative uncertainty of the transient measurements is 1 % at the 95 % level of confidence. This is the first report of thermal conductivity measurements made by two different methods in the same apparatus. The steady-state method is shown to complement normal transient measurements at low densities, particularly for fluids where the thermophysical properties at low densities are not known with high accuracy.

## 1. Introduction

The transient hot-wire system has been accepted widely as the most accurate technique for measuring the thermal conductivity of fluids over a wide range of physical states removed from the critical region. However, one of the drawbacks of this system is the need for increasingly larger corrections for the finite wire diameter and the outer boundary in the limit of zero fluid density. This effectively establishes a lower limit in pressure, at approximately 1 MPa for argon (corresponding to 28 kg m^−3^ in density), where the uncertainty in thermal conductivity increases dramatically. If transient measurements are made on gases below 1 MPa, the thermal conductivity is generally higher than the best theoretical estimates. Below 1 MPa, the linear region in the temperature rise vs the logarithm of time is greatly reduced or no longer exists for transient hot-wire measurements of gases. This curvature in the transient temperature rise is due to extremely large effects of the correction for finite physical properties of the wire at short times, and to the penetration of the transient thermal wave to the outer boundary at longer times, as the thermal diffusivity increases significantly in the limit of zero density. At low densities, the magnitudes of these corrections (comparable to the measured temperature rise itself) make it almost impossible to obtain an accurate mathematical description of the observed transient heat transfer in the hot-wire cells.

To overcome these difficulties, researchers often extrapolate measurements along an isotherm from higher densities to obtain the thermal conductivity of the dilute gas. The dilute-gas thermal-conductivity data obtained by such an extrapolation procedure have significantly more uncertainty than the data used in the extrapolation. Near the critical temperature the critical enhancement contributes significantly to the total thermal conductivity even at relatively low densities. This introduces curvature in thermal conductivity isotherms and makes the extrapolation to the dilute gas limit even more uncertain for isotherms near the critical temperature. Furthermore, the 1 MPa restriction makes the transient hot-wire instruments inappropriate for measuring thermal conductivity in the vapor phase at temperatures where the vapor pressure is below 1 MPa. Inconsistencies in dilute-gas thermal conductivities obtained by various researchers using transient hot wires have seriously weakened the credibility of the technique.

These problems increase the relative uncertainty at the level of 95 % confidence of the thermal conductivity obtained with the transient hot-wire technique from 0.3 % for measurements at higher densities to about 2 % at the lower densities, which are the focus of the present work. This 2 % relative uncertainty is comparable to the relative uncertainty of measurements obtained with accurate steady-state instruments. The largest uncertainty in steady-state measurements is due to fluid convection and this is known to decrease dramatically in the limit of zero density. Corrections to steady-state measurements actually decrease and become negligible in this dilute-gas region where transient measurements encounter their most serious difficulties.

At low densities the transient mode of heat transfer occurs at extremely short real times, where the wire heat-capacity correction is still quite large. This is because the thermal diffusivity of the gas is very large at low densities. This fast approach to steady state at low gas densities is an advantage for steady-state measurements using the same wire geometry. This paper examines the possibility of using the steady-state mode of operation to obtain the thermal conductivity of the dilute gas which is consistent with the higher density data obtained with the transient mode. This would allow any hot-wire instrument to operate in a transient mode at higher densities and in a steady-state mode down to the dilute-gas limit.

Measurements at low density have been made on argon gas to test this concept. Argon was selected because the dilute-gas value can be evaluated from the second-order Chapman-Enskog kinetic theory using the known pair-interaction potential. In addition, there are many accurate measurements of the thermal conductivity of argon available in this region using both steady-state and transient techniques. Each series of measurements was made over a wide range of applied powers and included a transient and a steady-state measurement at each power level. Both the transient and the steady-state measurements are compared with the best available predictions from kinetic theory and the other data from the literature. Agreement between measurements using both modes of operation demonstrates the validity of both techniques. Researchers with transient hot wire instruments can potentially select the optimum technique for a given fluid state.

**Fig. 1:**
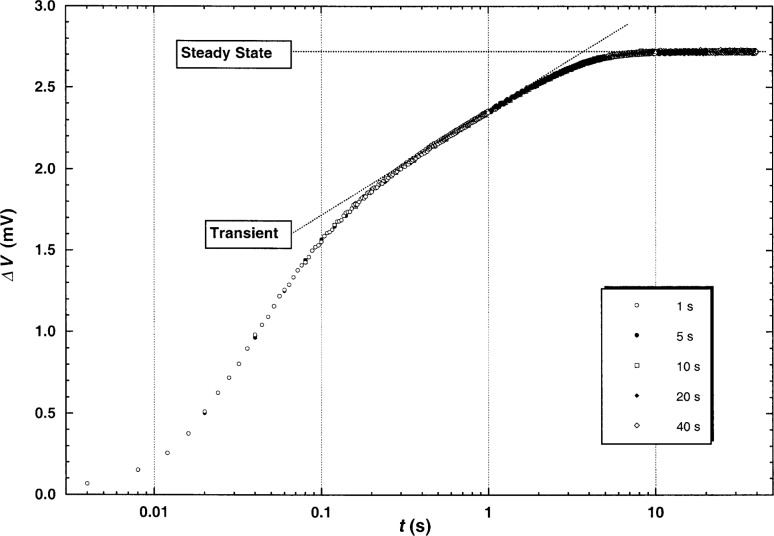
A composite of bridge imbalances from five experiments with different durations for argon at 1 MPa and 300 K. Both the rising transient region and the horizontal steady-state region are shown. Short time curvature is due to the finite wire diameter, while curvature between the transient and steady-state regions is due to penetration of the temperature gradient to the outer cell wall.

The transient hot-wire systems at NIST are completely described in previous papers [1–4]. The apparatus for high-temperature measurements [4] was used in the present work. The transient measurements were of one-second duration, as is typical in our previous measurements. The major change was modification of the data-acquisition system to operate in a steady-state mode, at times up to 40 s. A composite picture of the voltage rises across the Wheatstone bridge, obtained in five transient runs made at the same power level but with different experimental times, is shown in Fig. 1. Time is shown on a logarithmic scale, with the experimental times ranging from 1 s to 40 s. Two linear segments of the voltage rises are clearly visible. The transient thermal conductivity is obtained from the linear portion of finite slope which is proportional to the logarithm of time, while the steady-state thermal conductivity is calculated from the horizontal portion.

**Fig. 2:**
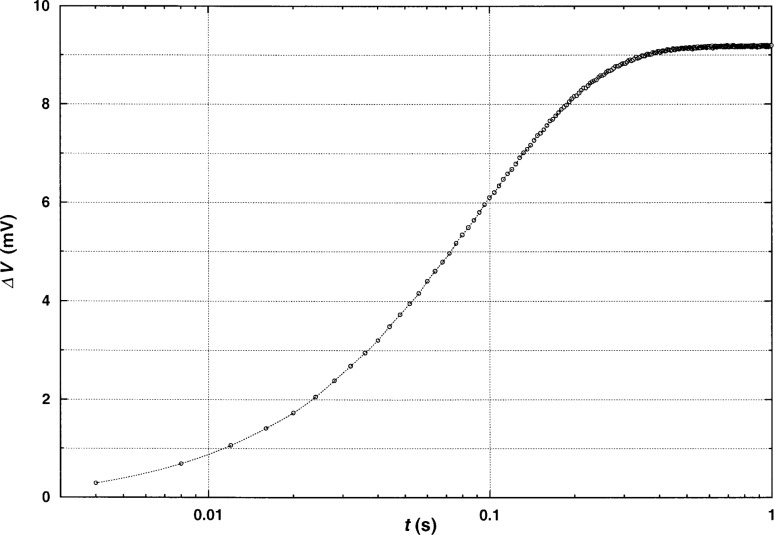
Bridge imbalance at conditions close to vacuum. Here, steady-state heat transfer is reached in less than 1 s and the transient region is almost nonexistent.

In Fig. 2, a typical voltage rise for a measurement in argon gas is shown, at a pressure below 1 kPa (mild vacuum), with an experimental duration of 1 s. This run would normally be evaluated as a transient experiment, the thermal conductivity being obtained from the apparent linear portion between 0.05 s and 0.15 s. There are two reasons to make measurements under these conditions. First, one can show that there is a sufficient section of a horizontal straight line, even at times below 1 s, to obtain a reliable result for steady-state conditions. Secondly, it may be possible to extract values for axial conduction or end effects. In addition, this extreme example shows exactly the origin of the problem with the transient experiment at low densities. The linear region in the temperature rise vs. the logarithm of time is extremely limited, degrading the accuracy of the resulting thermal conductivity data. In other words, a valid constant slope cannot be extracted from such a curve for temperature rise.

## 2. Transient Mode

Since transient measurements are increasingly unreliable as pressure decreases below 1 MPa, all assumptions made in the application of the theory of the instrument must be carefully examined. The theory for transient hot-wire measurements is well developed [5,6], although proper application of the theory requires significant care and judgment. The hot-wire cells are designed to approximate a transient line source as closely as possible, and deviations from this model are treated as corrections to the experimental temperature rise. The ideal temperature rise Δ*T*_id_ is given by

$$\Delta T_{\text{id}}=\frac{q}{4\pi \lambda }\left[\ln(t)+\ln\left(\frac{4a}{{r_{0}}^{2}C}\right)\right]=\Delta T_{W}+\sum_{i=1}^{10}\delta T_{i},$$ (1)

where *q* is the power applied divided by length, λ is the thermal conductivity of the fluid, *t* is the elapsed time, *a* = λ/*ρC_p_* is the thermal diffusivity of the fluid, *ρ* is the mass density of the fluid, *C_p_* is the isobaric heat capacity of the fluid, *r*_0_ is the radius of the hot wire, *C* = e*^γ^* = 1.781… is the exponential of Euler’s constant, Δ*T*_w_ is the measured temperature rise of the wire, and δ*T_i_* are corrections to account for deviations from ideal line-source conduction [5,6]. The two most significant corrections account for the finite radius of the wire and for penetration of the fluid temperature gradient to the outer cell wall. The finite wire radius produces the short-time temperature lag relative to the ideal model, as shown in Fig. 1. Penetration of the temperature gradient to the outer wall produces the transition from the linear transient region to the steady-state conduction mode, which is also shown in Fig. 1.

It is apparent in Eq. (1) that the thermal conductivity can be found from the slope of the ideal temperature rise as a function of the logarithm of elapsed time. The thermal diffusivity can be found from the intercept of this linear function. The uncertainty of the thermal conductivity at a level of confidence of 95 % is obtained from a linear fit of the ideal temperature rise data to Eq. (1) and is characterized by the parameter *STAT*. A *STAT* of 0.003, for example, corresponds to a reproducibility of 0.3 % for the reported thermal conductivity. The principal corrections to the ideal model account for the finite dimensions of the wire δ*T*_1_, penetration of the expanding thermal wave to the outer boundary δ*T*_2_, and thermal radiation δ*T*_5_. The relative magnitude of each correction depends on the fluid properties and the elapsed time in the experiment. In this work we apply the standard corrections [5,6], including the outer-boundary correction and the thermal-radiation correction for a transparent gas. The correction for the finite wire dimensions requires careful examination.

The hot wires have finite diameters and their specific heat introduces a temperature lag from the ideal line source model of Eq. (1). The temperature response of an infinitely long wire of finite radius *r*_0_ is given [5] by

$$\begin{array}{c} \Delta T_{\text{full}}(t,r)\\ =\frac{4q\lambda a}{\pi^{3}{r_{0}}^{3}}\int_{0}^{\infty }\frac{\left(1-\exp(-a_{W}u^{2}t)\right)J_{0}(ur)J_{1}(ur_{0})}{u^{4}\left(\phi^{2}(u)+\psi^{2}(u)\right)}du, \end{array}$$ (2)

where

$$\begin{array}{c} \phi (u)=\lambda_{w}a^{1/2}J_{1}(r_{0}u)J_{0}\left(r_{0}u\sqrt{a_{w}/a}\right)\\ -\lambda a_{w}^{0.5}J_{0}(r_{0}u)J_{1}\left(r_{0}u\sqrt{a_{w}/a}\right), \end{array}$$ (3)

and

$$\begin{array}{c} \psi (u)=\lambda_{w}a^{1/2}J_{1}(r_{0}u)Y_{0}\left(r_{0}u\sqrt{a_{w}/a}\right)\\ -\lambda a_{w}^{1/2}J_{0}(r_{0}u)Y_{1}\left(r_{0}u\sqrt{a_{w}/a}\right), \end{array}$$ (4)

where J*_n_* is the Bessel function of the first kind with order *n*, Y*_n_* is the Bessel function of the second kind with order *n*. In Eqs. (2)–(4) the thermal conductivity of the wire is λ_w_ and the thermal diffusivity of the wire is *a*_w_. Eqs. (2)–(4) are defined for any point in the wire, and it is the volume-averaged temperature from *r* = 0 to *r* = *r*_0_ which is required to correct our experimental observed rises in temperature. The resulting correction, δ*T*_1f_, for the finite wire dimension is

$$\delta T_{\text{lf}}=\int_{0}^{r_{0}}\frac{2\Delta T_{\text{full}}(r,t)r}{{r_{0}}^{2}}dr-\Delta T_{\text{id}}.$$ (5)

Although it is fairly simple to implement this full solution to correct the experimental temperature rise, it is the first-order expansion of this solution which has been recommended because of its simplicity [5,6]. This first-order expansion for a bare wire is [5,6]

$$\begin{array}{c} \delta T_{1}=\frac{q}{4\pi \lambda }[\frac{{r_{0}}^{2}\left({\left(\rho C_{p}\right)}_{w}-\rho C_{p}\right)}{2\lambda t}\ln\left(\frac{4at}{{r_{0}}^{2}C}\right)\\ -\frac{{r_{0}}^{2}}{2at}+\frac{{r_{0}}^{2}}{4a_{w}t}-\frac{\lambda }{2\lambda_{w}}]. \end{array}$$ (6)

**Fig. 3:**
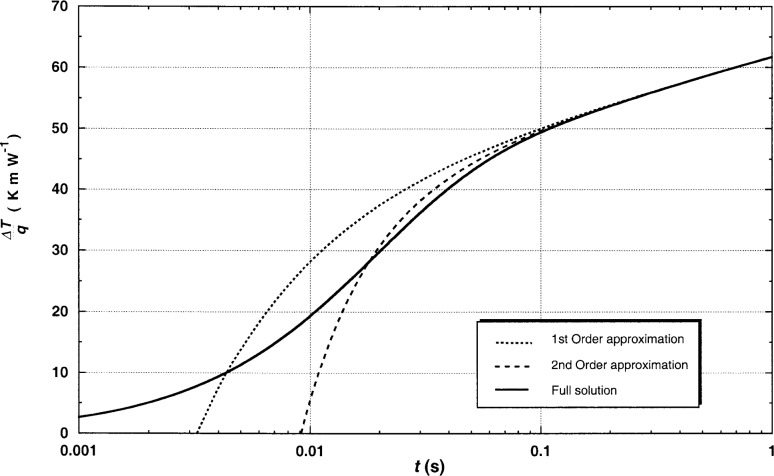
Calculated temperature rise for finite physical properties of the wire for argon gas at 300 K and 0.1 MPa using first-order and second-order approximations as well as the full integral solution.

Before an approximation such as Eq. (6) can be used, the truncation error for the case of our relatively large 12.7 μm platinum hot wire must be evaluated. Figure 3 shows the full solution for the transient temperature rise [Eqs. (2)–(5)], along with the first- [Eq. (6)] and second-order approximations [7], for the case of a 12.7 μm platinum hot wire in argon gas at 300 K and 0.1 MPa. It is apparent in Fig. 3 that the truncation error is quite Eq. (6) cannot be used to correct the data. Both the magnitude of the correction for physical properties of the finite wire and the associated truncation error can be minimized by using thinner hot wires. Table 1 shows how truncation corrections depend on the wire diameter for argon gas at 300 K and 0.1 MPa. Also included in Table 1 are the relative uncertainties in thermal conductivity resulting from a relative uncertainty of 10 % in the thermal diffusivity of the fluid.

In the limit of zero density, the transient thermal wave will penetrate to the outer boundary. The outer-boundary correction, which accounts for penetration of the transient heat pulse [5,6], is given by

$$\delta T_{2}=\frac{q}{4\pi \lambda }\left[\ln\left(\frac{4at}{r_{b}^{2}C}\right)+\sum_{\nu =1}^{\infty }\exp\left(\frac{-g_{\nu }at}{r_{b}^{2}}\right){\left[\pi Y_{0}(g_{\nu })\right]}^{2}\right],$$ (7)

where *g*_*ν*_ are the roots of J_0_ (*g*_*ν*_) = 0 and *r*_b_ is the radius of the outer boundary. In the limit of infinite time, Eq. (7) approaches the steady-state solution given below.

The final corrections, which must be considered because of their increasing significance at low densities, account for compression work δ*T*_3_ and radial convection δ*T*_4_. A recent analysis by Assael et al. [8] concludes that δ*T*_3_ and δ*T*_4_ must be considered simultaneously and that the previous analysis of Healy et al. [5] is in error. Based on the work of Assael et al. [8] we have set both δ*T*_3_ and δ*T*_4_ equal to zero in the present analysis.

At low densities, the thermal diffusivity of the fluid increases almost linearly with inverse pressure. Large corrections to the experimental temperature rise are required for both heat-capacity and outer-boundary effects because of this large thermal diffusivity. Any uncertainties in the wire diameter and the fluid properties used in these corrections become increasingly important at low densities. The full heat-capacity correction must be used to correct the measured temperature rises at low densities since this correction is so significant. The measurements at low density must be carefully examined to verify that the thermal wave has not reached the outer boundary since the thermal diffusivity increases so dramatically in this region.

**Fig. 4:**
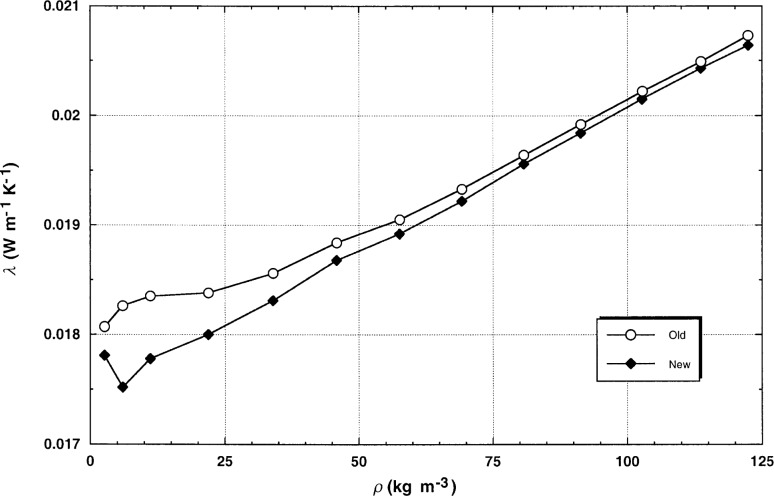
Transient results for argon at 300 K with the corrections applied in earlier papers as well as the revised corrections proposed herein. Results were averaged at each pressure.

Transient results obtained by use of the traditional corrections employed in earlier work [5,6] and the revised corrections as discussed above are shown for argon at 300 K in Fig. 4. The differences are primarily due to setting the compression work δ*T*_3_ equal to zero [8], use of the full heat-capacity correction of Eqs. (2)–(5), and careful restriction of the regression limits to exclude times where the outer-boundary correction δ*T*_2_ is significant. The results for all power levels were averaged at each pressure level in Fig. 4. It can be seen that the results at low densities are more linear in terms of density with the revised corrections (hook due to increasing contributions from the outer boundary correction), while the results at the higher densities are not changed appreciably. This linear dependence on density is expected from the kinetic theory of low-density gases.

## 3. Steady-State Mode

The working equation for the steady-state mode is based on a different solution of Fourier’s law but the geometry is still that of concentric cylinders. The solution can be found in standard texts for the case of constant thermal conductivity (see, for example, Reference [9], page 114). This equation can be solved for the thermal conductivity of the fluid λ;

$$\lambda =\frac{q\ln\left(\frac{r_{2}}{r_{1}}\right)}{2\pi (T_{1}-T_{2})},$$ (8)

where *q* is the applied power divided by length, *r*_2_ is the internal radius of the outer cylinder, *r*_1_ is the external radius of the inner cylinder (hot wire), and Δ*T* = (*T*_1_ − *T*_2_) is the measured temperature difference between the hot wire and its surrounding cavity.

For the concentric-cylinder geometry described above the total heat flux divided by length, *q*, remains constant and is not a function of the radial position. Assuming that the thermal conductivity is a linear function of temperature, such that λ = λ_0_(1 + *b_λ_T*), it can be shown that the measured thermal conductivity is given by λ = λ_0_(1 + *b_λ_*(*T*_1_ + *T*_2_)/2). Thus, the thermal conductivity that is measured corresponds to the value at the mean temperature of the inner and outer cylinders, where

$$\bar{T}=(T_{1}+T_{2})/2.$$ (9)

This assumption of a linear temperature dependence for the thermal conductivity is valid for experiments with small temperature rises. The density of the fluid assigned to the measured thermal conductivity is taken from an equation of state [10] using the temperature from Eq. (9) and the experimentally measured pressure.

**Fig. 5:**
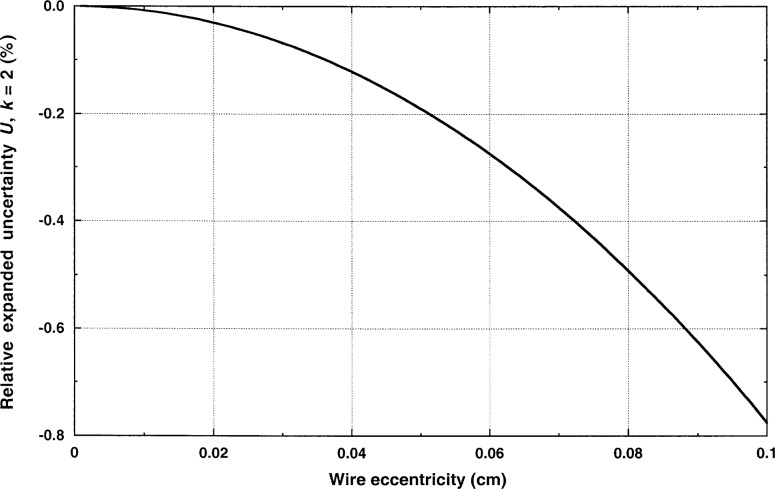
Error estimate for steady-state results due to the eccentricity of the hot wire. The thermal conductivity measurement is not sensitive to eccentricity for hot wires with small diameters.

Equation (8) assumes that the dimensions of the wire and cavity are well known and that the wire is perfectly concentric with the outer cylindrical cavity. The diameter of our wire is 13.14 µm and it is known with a relative uncertainty of 0.5 % at a level of confidence of 95 %. This uncertainty contributes a component of relative uncertainty of 0.07 % to the uncertainty of the measured thermal conductivity. Since it is nearly impossible to keep the wire perfectly concentric with the outer cavity, the uncertainty associated with the eccentricity of the wire with respect to the cavity must be assessed. For a wire that is eccentric, the thermal conductivity is given by

$$\begin{array}{c} \lambda =\frac{q}{2\pi (T_{1}-T_{2})}\\ \ln\left[\frac{\sqrt{{\left(r_{2}+r_{1}\right)}^{2}-b^{2}}+\sqrt{{\left(r_{2}-r_{1}\right)}^{2}-b^{2}}}{\sqrt{{\left(r_{2}+r_{1}\right)}^{2}-b^{2}}-\sqrt{{\left(r_{2}-r_{1}\right)}^{2}-b^{2}}}\right], \end{array}$$ (10)

where *b* is the distance between the wire’s axis and the axis of the outer cylinder. The eccentricity correction is shown in Fig. 5 for a 12.7 µm diameter wire in a 9 mm diameter cavity. The hot wires in the present cell are concentric with the cavity within 0.5 mm, so it is apparent from Fig. 5 that the relative uncertainty is about 0.2 % due to misalignment of the wire in the cavity. The combined relative uncertainty, due to both the diameter and eccentricity of the wire is 0.3 % in the measured thermal conductivity.

While 40 s may seem to be a very short time in comparison to normal steady-state measurements, it still allows the very small wires used in transient hot-wire systems to equilibrate in the gas phase. The time of the steady-state experiments is restricted to 40 s since the temperature of the cell wall *T*_1_ is assumed to be the initial cell temperature. Both transient and steady-state measurements of thermal conductivity should be made at several power levels. The thermal conductivity should be valid and free of convection if a plot of the measured values of thermal conductivity as a function of applied power is constant. The present measurements are made over a large range of applied powers, and the powers of the transient measurements overlap those used for the steady-state measurements as much as possible.

## 4. Data Reduction

Three isotherms were measured for gaseous argon at 300 K, 320 K, and 340 K. There were 13 to 14 different pressure levels covering a density range from 120 kg m^−3^ down to 2.4 kg m^−3^. At each pressure level, experimental results were collected at 7 to 11 different applied powers. Transient measurements were made for an experimental time of 1 s, while for the steady-state measurements the total elapsed time used was 40 s. In either case, 250 measurements of the bridge imbalance voltage were obtained. To elucidate the end effects in the experiment, a special series of runs were made using an additional digital voltmeter to measure directly across the long hot wire, the short hot wire, and the bridge. Finally, an abbreviated set of measurements was made for pressures below 1 kPa. In all, we made 883 measurements. The temperatures were measured on the International Practical Temperature Scale of 1968 (IPTS 68) but the effect of converting the temperatures to the International Temperature Scale of 1990 (ITS 90) on the reported thermal conductivity is less than 1 μW m^−1^ K^−1^.

**Fig. 6:**
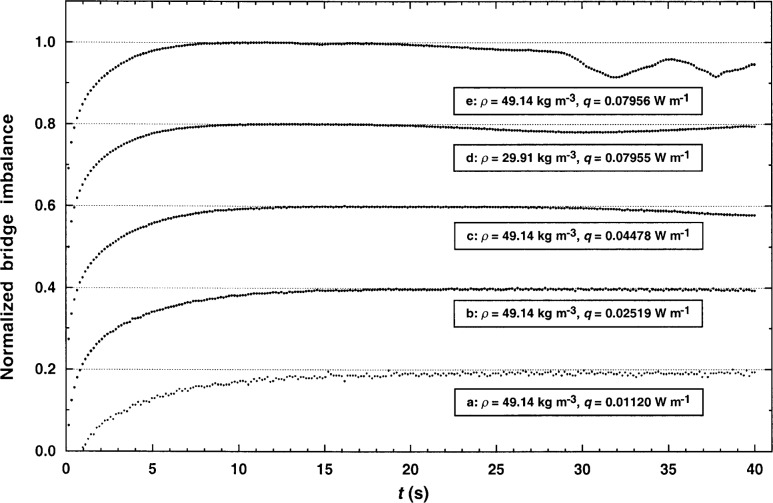
Typical voltage-rise profiles for steady-state measurements at *T* = 340 K. The increase in convection with the increase of applied power is shown in curves a through c. The increase in convection with increase in fluid density is shown in curves d and e.

The steady-state measurements required the development of a new data-analysis procedure. The rises in steady-state voltage as a function of time were always examined to select reliable measurements. Five typical profiles of the bridge imbalance, used to select the appropriate range of power levels, are shown in Fig. 6. Trace b in Fig. 6 is considered reliable since it is nearly horizontal after 20 s. In trace a, the power level is too low, so electronic noise is significant in the imbalance voltages. In traces c, d, and e the power levels are too large, so convection makes a visible contribution.

**Fig. 7:**
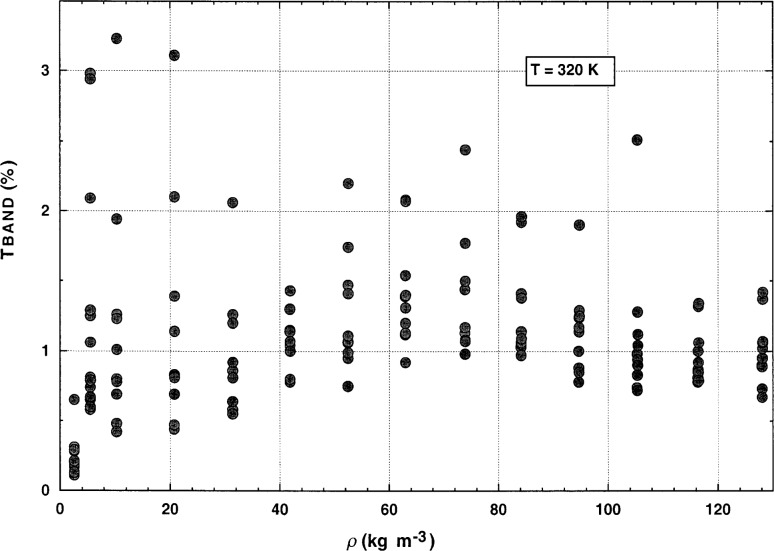
Temperature uncertainty, *TBAND*, for the 320 K isotherm of argon. Data with *TBAND* values greater that 2 % were considered invalid due to free-convection contributions.

The next step was to determine the experimental temperature rise Δ*T*. The experimental voltage rises were averaged over a time interval where they were nearly constant. To find the optimum time to begin the averaging, the last 50 bridge imbalance voltages were averaged to find a reference imbalance voltage. The actual average, *V*_ave_, is obtained by averaging the points from the first voltage, which is 0.5 % below this reference imbalance voltage up to the final data point. Solving the bridge equation with *V*_ave_ yields the change in resistance in the variable arms of the bridge. The resistance change is finally converted into Δ*T* using the calibration of the wire resistance as a function of temperature. The maximum and minimum values of the voltage rises were also obtained over the range averaged. The difference between them was expressed as a percentage of *V*_ave_, and is designated by the parameter *TBAND*. *TBAND* is a direct measure of the precision in Δ*T* at the level of 3 standard deviations. In Fig. 7, the values of *TBAND* are plotted for all of the steady-state measurements made near 320 K. A final selection of valid measurements was made by rejecting all points with a *TBAND* larger than 2 %. This is equivalent to rejecting those points that have voltage traces similar to traces c, d, and e in Fig. 6.

A correction for radiation was also applied to Δ*T*. The radiation correction for transparent fluids δ*T*_5_ was used as given in Ref. [4]. The maximum effect of this correction was 0.13 % at 340 K. Additional corrections have been considered by other authors for steady-state hot-wire systems (see for example Refs. [9,11]). These include corrections for temperature jump, end conduction in the wire, lead-wire conduction, and temperature rise in the outer wall. The temperature-jump correction does not apply because the present pressures are not low enough. The correction for end conduction in the wire and the lead-wire correction were found to be negligible in our experiments because a bridge with a compensating hot wire was used. A special series of measurements was made to determine the size of the end effects by directly measuring the temperature rise of each wire. Temperature gradients in the outer wall were considered negligible for our thick-walled pressure vessel. The primary platinum resistance thermometer (PRT) was mounted on the outside of the pressure vessel. The temperature *T*_ref_ of the PRT increased by about 30 mK for a series of measurements at a single pressure level. The temperature of the long hot wire, which is inside the cell, increased by a nearly identical amount. Since a measurement series normally consisted of about 20 measurements at time intervals 1 min apart, an average change of 1 mK per measurement was negligible in comparison to the measured Δ*T*, which was typically from 1 K to 4 K.

### 4.1 Free Convection

**Fig. 8:**
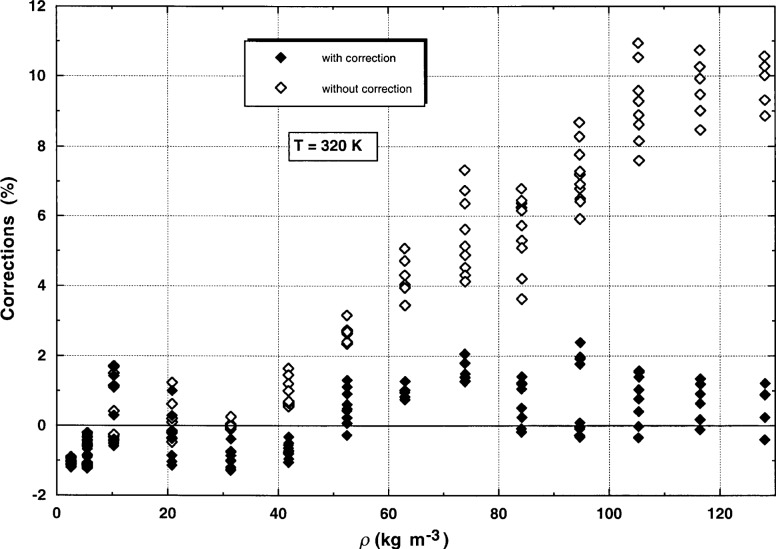
Correction of steady-state results for free convection along the 320 K isotherm. The baseline is the correlation of Younglove and Hanley [12].

Convection has always been a problem in measuring thermal conductivity; its onset has been associated with the Rayleigh number. One of the major advantages of the transient method is the ability to detect and avoid contributions from convection. The present measurements using the steady-state mode also show the evolution of convection very clearly. Figure 8 shows the deviations between the steady-state measurements near 320 K, calculated before application of the correction for convection, and the thermal conductivity surface for argon [12]. Since the diameter of the hot wires is comparable to the boundary-layer thickness for heat transfer, the standard engineering models for vertical flat plates are not applicable, and so an empirical expression was developed for the thin-wire geometry.

The dimensionless Rayleigh number is commonly used to characterize the onset of free convection. For a concentric-cylinder geometry, the Rayleigh number is given by

$$Ra=\frac{g_{c}{\left(r_{b}-r_{0}\right)}^{3}{\left(\frac{\partial \rho }{\partial T}\right)}_{P}\Delta T}{\eta a}$$ (11)

where *g*_c_ is the local acceleration of free fall, and is the fluid viscosity. The correction for natural or free convection was obtained from two equations given by Le Neindre and Tufeu [13] for a concentric-cylinder apparatus:

$$q=q_{\text{meas}}-q_{c},$$ (12)

and

$$\frac{q_{c}}{q}=\left(\frac{d}{720l}\right)Ra=K\cdot Ra,$$ (13)

where *q* is the applied power divided by length of Eq. (1)*q*_meas_ is the experimental heat flow determined from the measured voltage and current, *q*_c_ is the heat transfer by natural convection, *K* is a numerical apparatus constant, and *Ra* is the Rayleigh number. Le Neindre and Tufeu use a numerical constant of 720, but also values of *d*, the thickness of the fluid layer, and *l*, the length of the internal cylinder. For our system, both *d* and *l* are constant and our ratio of *d*/*l* is much larger than that of Le Neindre and Tufeu. Since the aspect ratio *d*/*l* is constant in our apparatus, it is incorporated into the experimentally determined apparatus constant *K* for our hot-wire cell. Equations (12) and (13) together give

$$\frac{q}{q_{\text{meas}}}=\frac{1}{1+K\cdot Ra}.$$ (14)

Next, Eq. (8) is applied twice, once for uncorrected conditions and once for corrected conditions. Forming a ratio we can solve for the corrected thermal conductivity

$$\lambda_{\text{corr}}=\left(\frac{q}{q_{\text{meas}}}\right)\lambda_{\text{meas}},$$ (15)

or with the use of Eq. (14),

$$\lambda_{\text{corr}}=\frac{1}{\left(1+K\cdot Ra\right)}\lambda_{\text{meas}}.$$ (16)

The best value for *K* in Eq. (16) was 1.8435 × 10^−6^ and was obtained by comparing the experimental points for each isotherm against a parabolic fit of the companion transient measurements. This procedure is justified because the deviations of the companion transient measurements from the thermal conductivity surface of argon are less than 1 % [12]. After applying this correction for free convection, Eq. (16), to all of the steady-state measurements, the resulting deviations are plotted in Fig. 8 for the 320 K isotherm. Figure 8 contains three different regions. For densities from 0 to 40 kg m^−3^, convection contributes very little to the measured conductivity, i.e., the corrections for convection are less than 1 %. In this region, the Rayleigh numbers range from near 0 to 17 000. For densities between 40 kg m^−3^ and 80 kg m^−3^ the corrections given by Eq. (16) gradually increase to about 5 %, while the Rayleigh numbers range up to 65 000. The correction is highly successful, as the band or range of measured values at each pressure level decreases. For steady-state thermal conductivities at densities above about 80 kg m^−3^, for which some of the Rayleigh numbers range well above 65 000, the corrections for simple natural convection increase to about 12 %. However, the uncertainty bands associated with the uncorrected thermal conductivities no longer decrease. The measured voltage rises suggest that there is flow turbulence in the cells at the larger power levels. This could be along either the short hot wire, the long hot wire, or both. Measurements associated with Rayleigh numbers greater than 70 000 have to be rejected, and are omitted from Fig. 8.

### 4.2 Results

**Fig. 9:**
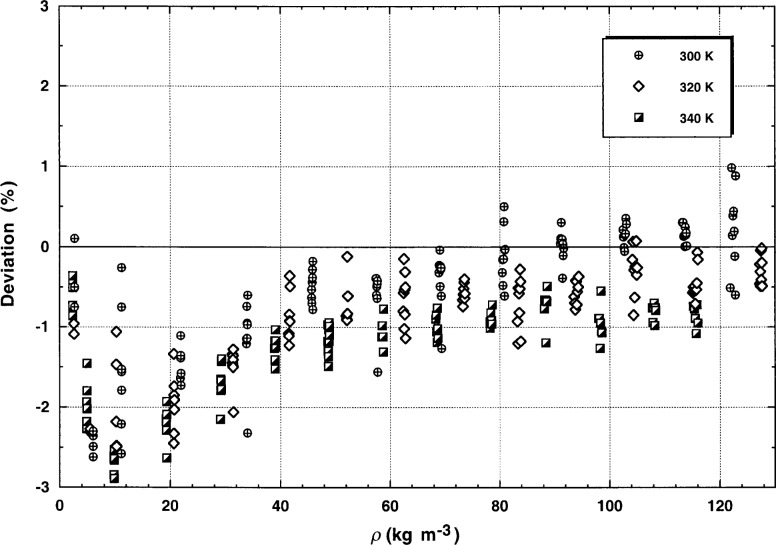
Transient thermal conductivity results for argon. The baseline is the correlation of Younglove and Hanley [12].

The transient measurements for all three isotherms are given in Table 2. All of the transient points with a *STAT* greater than 0.003 were eliminated; thus, 98 points remain at a nominal temperature of 300 K, 105 points remain at 320 K, and 102 points remain at 340 K. Figure 9 shows the deviations between all transient points and the thermal-conductivity surface of argon [12]. Based on Fig. 9 it can be concluded that, over the range of densities shown here, the deviations fit within a band of ±1 %. The problem with the transient measurements at low densities shows up clearly as a systematic deviation. However, as shown later, this systematic deviation must be ascribed to a difference in the dilute gas λ_0_ values used for the surface [12].

**Fig. 10:**
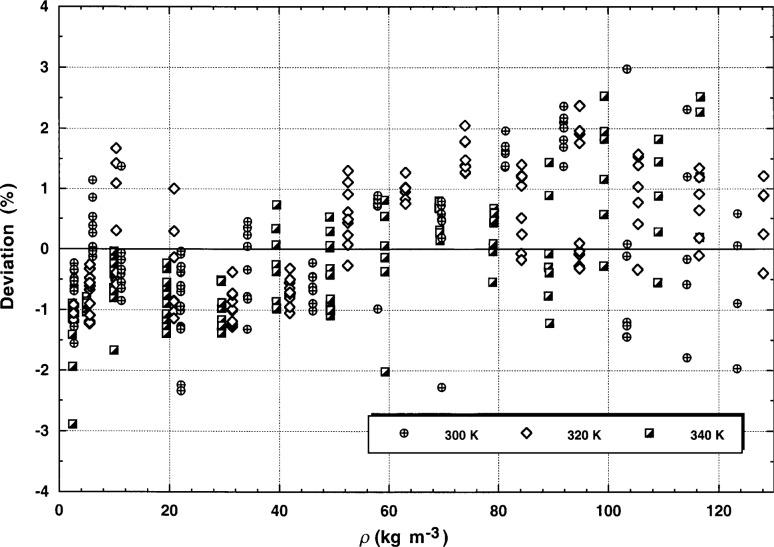
Steady-state thermal conductivity results for argon. The baseline is the correlation of Younglove and Hanley [12].

The steady-state measurements are listed in Table 3. As explained before, all steady-state results with *TBAND* greater than 2 % and all points with Rayleigh numbers greater than 70 000 were not considered. Thus, there are 104 points at a nominal temperature of 300 K, 120 points at 320 K, and 119 points at 340 K. The deviations between all steady-state points and the thermal-conductivity surface of argon [12] are plotted in Fig. 10. It can be concluded from Fig. 10 that over the range of densities shown, the deviations fit within a band of ±2 % at a level of confidence of 95 %. The scales of Figs. 9 and 10 are deliberately the same. Superimposing Figs. 9 and 10 shows that the systematic deviations at low densities are also seen in the steady-state measurements, which is why we ascribe this systematic deviation to a difference in the values for dilute gas thermal conductivity λ0. The agreement between the transient measurements and the steady-state results is quite good; the mean deviation between the two methods is 1 %.

**Fig. 11:**
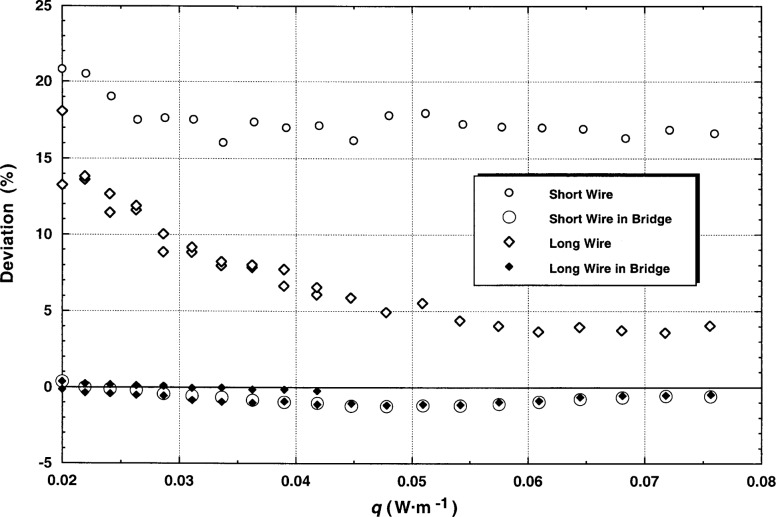
End effect ratios from “single wire” measurements to determine the effectiveness of end effect compensation by the bridge circuit. The baseline is the correlation of Younglove and Hanley [12].

The steady-state single-wire results are given in Table 4 under several different headings. The single-wire experiments are an attempt to measure the end effects in each wire. At the ends of each wire, heat is flowing from the wire ends to the wire supports. In addition, a part of the applied heat is flowing from the end of the wire through the fluid to the cell ends, a geometry quite different from the center portions of the wire. The lines in Table 4 are in pairs by run and point number. The first line is the regular or normal result calculated from the measured bridge imbalances, while the result on the second line uses the voltages measured directly across the individual hot wires. As a check the added voltmeter was also connected across the bridge. In this case the two lines given in Table 4 are virtually identical. Relative deviations of the data from the thermal conductivity surface of argon [12] are also provided in Table 4. These deviations are shown in Fig. 11 as a function of the applied power *q*. We see that for the short hot wire the thermal conductivity results are about 20 % above the normal steady-state bridge values, while for the long hot wire the deviation is around 4 % for the higher powers. An inspection of the single-wire voltage profiles indicates that turbulent convection (see trace c of Fig. 6) is first seen in the short-wire cell; however, it carries over into the full-bridge measurement. For the low-temperature system [1], using wire lengths of 0.05 m and 0.10 m, end effects of 8.3 % for the short wire and 4.8 % for the long wire were observed in nitrogen gas. The present wires have lengths of 0.05 m and 0.20 m, with end effects of 16 % and 4 %, respectively. For equivalent wire lengths (short wires), the present end effects are larger by about a factor of 2, quite reasonable given that the present steady-state measurements run considerably longer in time on a different gas.

The very last segment in Table 4 shows a series of runs made at a pressure considerably below ambient with the added voltmeter connected across the short hot wire. The exact pressure was difficult to determine because we did not have an appropriate pressure gage. We estimate that the pressure must be between 2 Pa and 1300 Pa, somewhere between the limit of the forepump and the rather approximate reading of the regular pressure gage. We can be certain that these measurements fall into the Knudsen region where the thermal conductivity is proportional to pressure. The deviations for the two highest power levels in this series fall between the ones for the long and the short hot wires. We might expect the end effects to be smaller under these conditions because the heat flow from the wire ends to the cell ends is reduced.

It is shown that for all conditions, except perhaps turbulent convection in either cell, having the short hot wire in the bridge insures sufficient compensation for end effects. This seems to be in agreement with the conclusions of Taxis and Stephan [7]. Finally, the steady-state results, the first line of each pair, are independent of applied power, an excellent verification of the key requirement that the results are free from the influence of natural convection.

### 4.3 Analysis

Argon was selected as the test fluid because the dilute-gas thermal conductivity λ0 and the first density correction λ_1_ = (*∂λ*/*∂λρ*)_T_ are well known from Chapman-Enskog theory, as is the pair interaction potential for argon. The present results must be compared with the values derived from theory to validate the technique. To make the analysis easier, the large number of points was reduced by averaging as follows. All of the results were shifted from their experimental temperatures to the even temperatures of 300 K, 320 K, and 340 K using the thermal conductivity surface for argon [12]. The mean of this adjustment is −0.35 %, the maximum is −1.25 %. The next step is to average the results for the various power levels at each pressure. This step gives us 13 or 14 points per isotherm. Theory indicates that the thermal conductivity is a nearly linear function of density at low densities. Since values are now available, measured by two different methods in the same apparatus, it is reasonable to combine both transient and steady-state values into one result. Thus the final step is to obtain averaged straight lines for each isotherm from the experimental results. These averaged straight lines become the basis for deviation plots to assess the accuracy of the present steady-state and transient results.

The averaged thermal conductivities adjusted to nominal isotherm temperatures are given in Table 5 along with the deviations of these values from the straight lines. From Table 5 it is easy to establish that the mean difference between transient and steady-state measurements is about 1 %, with the steady-state values nearly always higher. While transient and steady-state values agree to within their combined uncertainties, we cannot exclude the possibility that a systematic difference of about 1 % may exist between the two methods. Our straight-line intercepts are the values of the dilute-gas thermal conductivity λ0 and the slopes are values of the first density correction λ1. The coefficients of the lines with their calculated expanded uncertainty at a level of uncertainty of 95 % are given in Table 6.

The reason that we have used the thermal conductivity surface of Younglove and Hanley [12] in this paper for comparisons, etc., rather than some of the other possible choices, will now become clear. The dilute-gas thermal conductivities of the Younglove and Hanley model [12] are equivalent to the theoretically derived values of Kestin et al. [14]. We conclude from Table 6 that our dilute-gas thermal conductivities are lower than the theoretical values of Kestin et al. [14] as well as those of Aziz [15]. However, they appear to be in better agreement with those of Aziz. Our first density corrections are seen to depend slightly on temperature, unlike the theoretical results, which are constant. They appear to be in better agreement with the values of Rainwater and Friend [16, 17] than with those of Bich and Vogel [18]. Good agreement is found with the previous work using the NIST low temperature instrument [19].

**Fig. 12:**
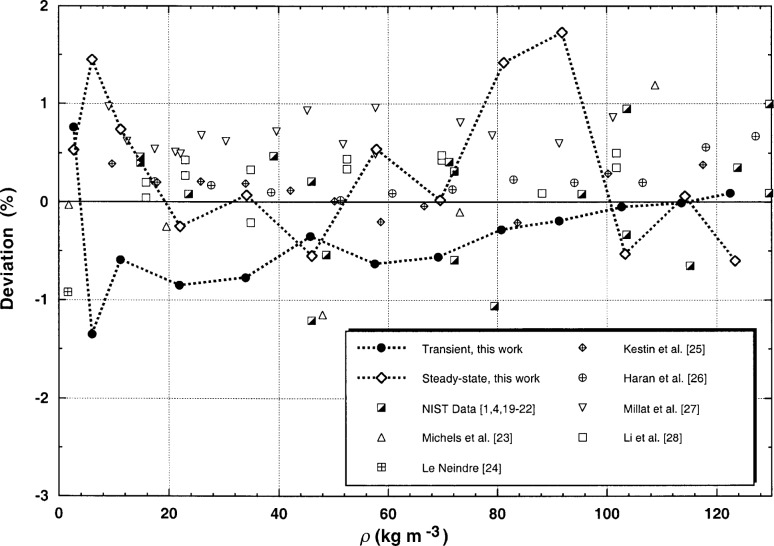
Deviations of the present results and those of other authors from the line average of the present transient and steady-state results for argon at 300 K.

Finally, in Fig. 12 the present results are compared with our earlier ones [1,4,19–22], and with those of other authors [23–28] for a temperature of 300 K, where the baseline is the present least-squares fitted line (coefficients in Table 6). The present results are connected by lines to set them off from the others. All other results were shifted to a temperature of 300 K by using the thermal-conductivity surface of [12]. The largest shift is around 2.3 %, because a few of the original experimental temperatures are as high as 308 K. Figure 12 shows that the present results, including the new steady-state data, are in good agreement with our earlier results [1,4,19–22]. Figure 12 also shows that all of the values assembled here, which include those from transient experiments, steady-state concentric cylinders [24], and steady-state parallel plate systems [23], agree to within 1 %, a truly remarkable result. Comparisons made at the other two temperatures, 320 and 340 K, are quite similar to Fig. 12.

### 4.4 Uncertainty

The measurements were deliberately made over a wide range of power levels. For both transient and steady-state results those power levels which were either too small or too large were eliminated. For power levels that are too low, the bridge imbalance becomes comparable to the background noise level and there is significant uncertainty in the measured temperature rises. In general the instruments require a temperature rise of at least 2.5 K to obtain accurate transient results (*STAT* < 0.003). With power levels that are too high, curvature is found in the relation for Δ*T* vs ln(*t*) for the transient measurements, as is typical of convection. The onset of natural convection is also observed in the steady-state measurements as a time-varying steady-state temperature rise. Imposing certain limits on the experimental uncertainty parameter—a maximum *STAT* of 0.003 for the transient points and a maximum of 2 % in *TBAND* for the steady-state points—seem to be appropriate restrictions. The uncertainty in transient thermal conductivity data increases at densities below 28 kg m^−3^ (*p* = 1 MPa). For valid steady-state measurements of the thermal conductivity, the Rayleigh number must be less than 70 000. With these restrictions it is found that the relative expanded uncertainty of the transient thermal conductivity is 1 % (*k* = 2, see Fig. 9), the uncertainty of the steady-state thermal conductivity is 2 % (*k* = 2, see Fig. 10), while the agreement between the two methods is 1 % (see Table 5). The overall agreement between our present results, our earlier transient results and the results of many other authors is a truly remarkable 1 % (see Fig. 12).

The steady-state results have a greater uncertainty than the transient ones. This can be attributed to a number of factors. First, the steady-state experiment requires an accurate measurement of the temperature rise Δ*T* quite similar to the measurement of thermal diffusivity in the transient hot-wire system [20,21]. Second, the steady-state measurement requires accurate determination of the cell geometry: wire radius, cavity radius, eccentricity. Due to the constraints imposed by the onset of convection, the valid temperature rises for steady-state measurements are half those of the transient ones. Table 7 shows the mean temperature rises for both modes of operation. This may serve as a guide for operating hot-wire cells in either the transient or steady-state mode.

## 5. Summary

It has been demonstrated that the thermal conductivity of argon can be measured with a relative expanded uncertainty (*k* = 2) of 2 %, using a transient hot-wire system operating in an absolute steady-state mode. The bridge arrangement used in this experiment provides sufficient compensation, eliminating the problem of end effects for both transient and steady-state measurements. The selection of valid steady-state results is based on the shape of the curve of measured voltage rises, on the size of the error in the temperature rise, *TBAND*, and on the magnitude of the Rayleigh number. Since the fluid gap is quite large in transient hot-wire cells, the steady-state mode is restricted to the low-density gas. The use of the absolute steady-state mode requires no information on the thermophysical properties of the fluid of interest. This is a significant advantage for the measurement of thermal conductivities in the vapor phase of refrigerants where the fluid properties are not known well enough to obtain accurate corrections for transient hot-wire measurements. From a fundamental point of view it can contribute to the determination of accurate pair-interaction potentials for diatomic molecules, especially the long-range weak interactions, a very active research field, by measuring the thermal conductivity of low-temperature vapors.

## Figures and Tables

**Table 1:** Uncertainties due to corrections for finite wire diameter for argon gas at 300 K and 0.1 MPa. First, differences are examined between the actual heat transfer from a finite diameter wire and the first and second order series approximations of this solution for various wire diameters. Second, the effect of a 10 % error in the thermal diffusivity *a* used for finite wire diameter correction is calculated for various wire diameters. This correction can introduce significant errors in thermal conductivity measurements with large wires if the thermal diffusivity or wire diameter is not well known.

Fluid	Wire diameter (μm)	Ideal slope	Slope after 1st order correction is applied	Slope after 2nd order correction is applied	Relative deviation in λ after 1st order correction	Relative deviation in λ after 2nd order correction
Mathematical approximation errors						

Argon	7.0	4.445669	4.451398	4.446015	−0.12 %	−0.01 %
Argon	12.5	4.445669	4.504379	4.456487	−1.32 %	−0.24 %
Argon	25.0	4.445669	5.508840	4.952375	−23.91 %	−11.40 %

Fluid	Wire diameter (μm)	Ideal slope	Apparent slope with a − 10 % error in thermal diffusivity	Apparent slope with a +10 % error in thermal diffusivity	Relative deviation in λ with a − 10 % error in thermal diffusivity	Relative deviation in λ with a +10 % error in thermal diffusivity

Thermal conductivity uncertainty associated with 10 % uncertainty in fluid thermal diffusivity						

Argon	7.0	4.445669	4.444565	4.446668	−0.03 %	+0.02 %
Argon	12.5	4.445669	4.441077	4.449814	−0.10 %	+0.09 %
Argon	25.0	4.445669	4.398395	4.487762	−1.06 %	+0.94 %

**Table 2:** Thermal conductivity of argon, transient method

Point no.	*p* (MPa)	*ρ* (kg m^−3^)	*T*_exp_ (K)	λ_exp_ (W m^−1^ K^−1^)	*STAT*	Relative dev. (%)	*q* (W m^−1^)
Nominal temperature 300 K							

3001	7.4440	122.916	301.662	0.02092	0.002	0.88	0.03941
3002	7.4441	122.832	301.836	0.02062	0.003	−0.60	0.04485
3003	7.4440	122.736	302.022	0.02073	0.001	−0.12	0.05063
3004	7.4441	122.636	302.231	0.02080	0.001	0.19	0.05676
3005	7.4442	122.525	302.454	0.02086	0.001	0.44	0.06324
3006	7.4438	122.409	302.682	0.02086	0.001	0.38	0.07007
3007	7.4440	122.297	302.914	0.02082	0.001	0.14	0.07725
3008	7.4441	122.169	303.178	0.02101	0.001	0.98	0.08479
3010	7.4436	121.893	303.728	0.02072	0.001	−0.51	0.10090
3022	6.9077	113.988	301.325	0.02049	0.003	0.01	0.02960
3023	6.9078	113.916	301.488	0.02053	0.003	0.17	0.03433
3024	6.9079	113.836	301.667	0.02053	0.002	0.15	0.03941
3025	6.9076	113.744	301.853	0.02055	0.002	0.24	0.04484
3026	6.9076	113.652	302.061	0.02051	0.001	0.00	0.05062
3027	6.9077	113.560	302.270	0.02055	0.001	0.14	0.05676
3028	6.9078	113.460	302.495	0.02056	0.001	0.13	0.06324
3029	6.9078	113.348	302.744	0.02061	0.001	0.30	0.07007
3030	6.9078	113.237	302.994	0.02062	0.001	0.30	0.07726
3043	6.2650	103.038	301.530	0.02027	0.003	0.28	0.03433
3044	6.2650	102.962	301.714	0.02029	0.002	0.35	0.03942
3045	6.2651	102.886	301.910	0.02026	0.002	0.16	0.04485
3046	6.2651	102.806	302.110	0.02027	0.001	0.15	0.05063
3047	6.2651	102.714	302.333	0.02024	0.001	−0.06	0.05676
3048	6.2652	102.622	302.566	0.02026	0.001	−0.01	0.06325
3049	6.2652	102.527	302.805	0.02029	0.001	0.12	0.07008
3050	6.2652	102.423	303.069	0.02032	0.001	0.21	0.07727
3062	5.5849	91.645	301.383	0.01991	0.003	−0.02	0.02961
3063	5.5849	91.581	301.557	0.01990	0.002	−0.11	0.03434
3064	5.5851	91.521	301.737	0.01994	0.002	0.03	0.03942
3065	5.5849	91.445	301.942	0.01987	0.002	−0.39	0.04485
3066	5.5851	91.377	302.150	0.01997	0.002	0.09	0.05063
3067	5.5850	91.293	302.377	0.01998	0.001	0.09	0.05676
3068	5.5848	91.201	302.628	0.02003	0.002	0.30	0.06325
3069	5.5848	91.121	302.859	0.02001	0.001	0.10	0.07008
3070	5.5849	91.034	303.121	0.02001	0.001	0.04	0.07727
3082	4.9512	81.011	301.407	0.01965	0.003	−0.03	0.02961
3083	4.9514	80.959	301.589	0.01966	0.002	−0.04	0.03434
3084	4.9514	80.899	301.776	0.01956	0.002	−0.61	0.03942
3085	4.9516	80.839	301.980	0.01978	0.002	0.50	0.04485
3086	4.9517	80.775	302.198	0.01976	0.001	0.31	0.05063
3087	4.9517	80.707	302.427	0.01968	0.001	−0.15	0.05677
3088	4.9518	80.631	302.669	0.01962	0.001	−0.48	0.06325
3089	4.9519	80.555	302.926	0.01970	0.001	−0.16	0.07008
3090	4.9519	80.475	303.193	0.01968	0.001	−0.32	0.07727
3102	4.2568	69.414	301.448	0.01914	0.003	−1.27	0.02961
3103	4.2569	69.370	301.624	0.01928	0.002	−0.61	0.03434
3104	4.2569	69.318	301.821	0.01935	0.002	−0.26	0.03942
3105	4.2570	69.266	302.025	0.01936	0.002	−0.28	0.04485
3106	4.2570	69.206	302.258	0.01938	0.001	−0.24	0.05063
3107	4.2572	69.150	302.495	0.01934	0.001	−0.49	0.05677
3108	4.2569	69.082	302.739	0.01944	0.001	−0.04	0.06325
3109	4.2570	69.014	303.009	0.01942	0.001	−0.23	0.07009
3110	4.2572	68.946	303.284	0.01941	0.001	−0.32	0.07728
3123	3.5548	57.757	301.495	0.01883	0.003	−1.56	0.02961
3124	3.5545	57.713	301.678	0.01905	0.002	−0.42	0.03434
3125	3.5546	57.669	301.892	0.01902	0.002	−0.64	0.03942
3126	3.5546	57.625	302.108	0.01906	0.002	−0.46	0.04485
3127	3.5546	57.577	302.335	0.01907	0.001	−0.49	0.05064
3128	3.5547	57.529	302.581	0.01906	0.001	−0.60	0.05677
3129	3.5547	57.473	302.836	0.01911	0.001	−0.39	0.06326
3130	3.5545	57.413	303.112	0.01912	0.001	−0.39	0.07009
4003	2.8395	45.944	301.638	0.01883	0.003	−0.18	0.02960
4004	2.8397	45.916	301.831	0.01873	0.002	−0.78	0.03433
4005	2.8397	45.880	302.047	0.01881	0.002	−0.38	0.03941
4006	2.8397	45.844	302.264	0.01884	0.002	−0.28	0.04484
4007	2.8397	45.804	302.508	0.01882	0.001	−0.44	0.05063
4008	2.8397	45.764	302.765	0.01879	0.001	−0.70	0.05676
4009	2.8398	45.720	303.036	0.01883	0.001	−0.53	0.06324
4010	2.8397	45.673	303.324	0.01883	0.001	−0.63	0.07007
4023	2.1132	34.056	301.681	0.01850	0.003	−0.60	0.02962
4024	2.1132	34.032	301.896	0.01844	0.003	−0.97	0.03435
4025	2.1131	34.004	302.124	0.01821	0.002	−2.32	0.03943
4026	2.1130	33.976	302.346	0.01846	0.002	−0.95	0.04487
4027	2.1131	33.948	302.592	0.01844	0.001	−1.14	0.05065
4028	2.1132	33.916	302.857	0.01853	0.001	−0.74	0.05679
4029	2.1131	33.880	303.141	0.01847	0.001	−1.15	0.06328
4030	2.1132	33.848	303.444	0.01847	0.001	−1.21	0.07011
4045	1.3696	21.963	301.960	0.01808	0.002	−1.58	0.03435
4046	1.3696	21.947	302.195	0.01807	0.002	−1.73	0.03943
4047	1.3696	21.927	302.446	0.01815	0.002	−1.36	0.04487
4048	1.3697	21.907	302.709	0.01820	0.001	−1.11	0.05065
4049	1.3697	21.888	302.995	0.01817	0.001	−1.39	0.05679
4050	1.3697	21.864	303.298	0.01814	0.001	−1.64	0.06327
4084	0.7010	11.197	301.997	0.01788	0.003	−1.53	0.03435
4085	0.7012	11.189	302.256	0.01803	0.003	−0.75	0.03943
4086	0.7010	11.177	302.533	0.01813	0.003	−0.26	0.04487
4087	0.7010	11.165	302.796	0.01788	0.002	−1.79	0.05065
4088	0.7011	11.157	303.109	0.01793	0.002	−1.56	0.05679
4089	0.7011	11.145	303.417	0.01777	0.002	−2.58	0.06327
4090	0.7011	11.134	303.762	0.01785	0.003	−2.21	0.07011
4107	0.3782	6.024	302.352	0.01763	0.003	−2.49	0.03943
4108	0.3784	6.020	302.636	0.01768	0.002	−2.30	0.04487
4109	0.3784	6.016	302.930	0.01768	0.002	−2.36	0.05065
4110	0.3783	6.008	303.254	0.01765	0.002	−2.62	0.05679
4127	0.1684	2.681	302.206	0.01786	0.003	−0.75	0.03435
4128	0.1686	2.681	302.500	0.01792	0.003	−0.50	0.03943
4129	0.1686	2.677	302.817	0.01794	0.003	−0.51	0.04487
4130	0.1686	2.673	303.142	0.01806	0.003	0.10	0.05065

Nominal temperature 320 K							

5001	8.3272	127.678	321.430	0.02186	0.002	−0.49	0.04215
5002	8.3273	127.630	321.533	0.02193	0.002	−0.20	0.04501
5003	8.3273	127.590	321.617	0.02197	0.002	−0.02	0.04796
5004	8.3273	127.542	321.720	0.02189	0.002	−0.41	0.05100
5005	8.3275	127.494	321.822	0.02188	0.001	−0.47	0.05414
5006	8.3273	127.442	321.927	0.02198	0.001	−0.05	0.05737
5007	8.3275	127.394	322.036	0.02188	0.001	−0.49	0.06069
5008	8.3277	127.346	322.145	0.02193	0.001	−0.31	0.06411
5009	8.3275	127.286	322.262	0.02195	0.001	−0.22	0.06762
5010	8.3275	127.234	322.379	0.02190	0.001	−0.46	0.07123
5021	7.5805	116.005	321.557	0.02162	0.002	−0.16	0.04501
5022	7.5807	115.925	321.760	0.02164	0.002	−0.07	0.05100
5023	7.5808	115.837	321.967	0.02157	0.001	−0.45	0.05737
5024	7.5808	115.745	322.187	0.02157	0.001	−0.53	0.06411
5025	7.5808	115.645	322.424	0.02157	0.001	−0.54	0.07123
5026	7.5809	115.542	322.675	0.02155	0.001	−0.71	0.07871
5027	7.5812	115.438	322.931	0.02161	0.001	−0.49	0.08658
5028	7.5811	115.322	323.204	0.02160	0.001	−0.55	0.09482
5029	7.5812	115.202	323.490	0.02163	0.001	−0.50	0.10343
5030	7.5812	115.078	323.792	0.02162	0.001	−0.57	0.11241
5041	6.8721	104.995	321.578	0.02130	0.002	−0.26	0.04501
5042	6.8720	104.915	321.785	0.02129	0.001	−0.35	0.05100
5043	6.8720	104.836	321.999	0.02139	0.001	0.07	0.05737
5044	6.8721	104.744	322.246	0.02134	0.001	−0.24	0.06411
5045	6.8721	104.652	322.484	0.02135	0.001	−0.25	0.07122
5046	6.8721	104.560	322.735	0.02128	0.001	−0.63	0.07871
5047	6.8721	104.460	323.002	0.02136	0.001	−0.29	0.08658
5048	6.8721	104.356	323.279	0.02125	0.001	−0.85	0.09481
5049	6.8722	104.248	323.571	0.02146	0.001	0.06	0.10343
5050	6.8721	104.128	323.884	0.02143	0.001	−0.16	0.11241
5061	6.1871	94.369	321.598	0.02100	0.002	−0.37	0.04500
5062	6.1870	94.297	321.809	0.02099	0.002	−0.50	0.05099
5064	6.1869	94.133	322.288	0.02100	0.001	−0.56	0.06410
5065	6.1869	94.054	322.526	0.02097	0.001	−0.72	0.07122
5066	6.1868	93.970	322.773	0.02104	0.001	−0.43	0.07871
5067	6.1868	93.882	323.047	0.02106	0.001	−0.42	0.08657
5068	6.1867	93.782	323.335	0.02100	0.001	−0.78	0.09481
5069	6.1866	93.682	323.629	0.02103	0.001	−0.70	0.10342
5070	6.1866	93.578	323.942	0.02106	0.001	−0.62	0.11241
5082	5.5060	83.859	321.512	0.02057	0.002	−1.18	0.04215
5083	5.5060	83.803	321.705	0.02073	0.002	−0.43	0.04795
5084	5.5061	83.735	321.934	0.02077	0.001	−0.28	0.05414
5085	5.5061	83.667	322.164	0.02073	0.001	−0.52	0.06069
5086	5.5061	83.587	322.430	0.02068	0.002	−0.82	0.06762
5087	5.5061	83.515	322.685	0.02074	0.001	−0.58	0.07492
5088	5.5061	83.435	322.959	0.02077	0.001	−0.51	0.08260
5089	5.5061	83.352	323.250	0.02064	0.001	−1.21	0.09065
5090	5.5061	83.268	323.532	0.02071	0.001	−0.93	0.09908
5103	4.8431	73.672	321.333	0.02043	0.003	−0.58	0.03672
5104	4.8431	73.624	321.518	0.02042	0.002	−0.64	0.04215
5105	4.8432	73.572	321.728	0.02048	0.002	−0.40	0.04796
5106	4.8431	73.512	321.960	0.02048	0.002	−0.44	0.05414
5107	4.8432	73.452	322.198	0.02048	0.001	−0.52	0.06069
5108	4.8431	73.388	322.449	0.02048	0.001	−0.59	0.06762
5109	4.8431	73.321	322.712	0.02046	0.001	−0.74	0.07492
5110	4.8431	73.249	323.000	0.02049	0.001	−0.66	0.08260
5122	4.1373	62.842	321.147	0.02004	0.003	1.14	0.03166
5123	4.1373	62.798	321.339	0.02018	0.003	−0.50	0.03672
5124	4.1374	62.758	321.541	0.02023	0.002	−0.31	0.04215
5125	4.1374	62.706	321.776	0.02013	0.002	−0.84	0.04796
5126	4.1375	62.658	322.005	0.02021	0.002	−0.53	0.05414
5127	4.1375	62.603	322.266	0.02012	0.002	−1.02	0.06069
5128	4.1375	62.547	322.531	0.02031	0.001	−0.15	0.06762
5129	4.1375	62.491	322.791	0.02023	0.001	−0.57	0.07492
5130	4.1375	62.431	323.081	0.02020	0.001	−0.80	0.08260
5143	3.4551	52.320	321.388	0.01992	0.003	−0.61	0.03672
5145	3.4549	52.240	321.841	0.01989	0.002	−0.83	0.04795
5146	3.4550	52.200	322.077	0.02004	0.002	−0.12	0.05413
5147	3.4550	52.156	322.325	0.01990	0.001	−0.91	0.06069
5148	3.4546	52.104	322.584	0.01991	0.001	−0.90	0.06762
5149	3.4548	52.052	322.889	0.01994	0.001	−0.86	0.07492
5150	3.4549	52.000	323.191	0.01995	0.001	−0.86	0.08259
5163	2.7632	41.770	321.224	0.01969	0.003	−0.49	0.03165
5164	2.7631	41.738	321.429	0.01961	0.003	−0.93	0.03671
5165	2.7632	41.706	321.678	0.01973	0.002	−0.36	0.04214
5166	2.7633	41.678	321.896	0.01964	0.002	−0.92	0.04795
5167	2.7633	41.642	322.159	0.01966	0.002	−0.84	0.05413
5168	2.7632	41.602	322.422	0.01960	0.002	−1.23	0.06068
5169	2.7633	41.566	322.688	0.01964	0.001	−1.11	0.06761
5170	2.7633	41.526	322.985	0.01966	0.001	−1.08	0.07491
5193	2.0865	31.455	321.287	0.01916	0.003	−2.06	0.03165
5194	2.0865	31.435	321.494	0.01932	0.003	−1.28	0.03671
5195	2.0864	31.411	321.726	0.01929	0.002	−1.50	0.04215
5196	2.0866	31.387	321.970	0.01932	0.002	−1.41	0.04795
5197	2.0865	31.359	322.228	0.01934	0.002	−1.36	0.05413
5198	2.0865	31.331	322.499	0.01933	0.001	−1.48	0.06068
5199	2.0863	31.299	322.791	0.01935	0.001	−1.45	0.06761
5200	2.0863	31.267	323.096	0.01938	0.001	−1.34	0.07491
5214	1.3800	20.729	321.581	0.01897	0.003	−1.91	0.03671
5215	1.3798	20.713	321.822	0.01902	0.002	−1.74	0.04214
5216	1.3798	20.697	322.075	0.01898	0.003	−2.03	0.04795
5217	1.3798	20.677	322.346	0.01902	0.002	−1.85	0.05412
5218	1.3800	20.661	322.636	0.01895	0.001	−2.33	0.06068
5219	1.3800	20.641	322.948	0.01915	0.002	−1.34	0.06760
5220	1.3800	20.617	323.267	0.01895	0.001	−2.45	0.07490
5235	0.6849	10.251	321.919	0.01867	0.003	−2.49	0.04214
5236	0.6849	10.239	322.206	0.01894	0.003	−1.06	0.04794
5237	0.6849	10.231	322.494	0.01870	0.002	−2.48	0.05412
5239	0.6849	10.211	323.147	0.01891	0.002	−1.47	0.06760
5240	0.6849	10.203	323.465	0.01880	0.001	−2.18	0.07490
5259	0.3678	5.485	322.625	0.01864	0.002	−2.27	0.05412
5260	0.3679	5.481	322.970	0.01865	0.002	−2.31	0.06067
5277	0.1732	2.581	322.807	0.01881	0.002	−1.09	0.05412
5278	0.1734	2.577	323.722	0.01885	0.002	−1.09	0.07120
5279	0.1733	2.565	324.762	0.01893	0.002	−0.96	0.09062

Nominal temperature 340 K							

6001	8.1063	112.607	342.889	0.02259	0.001	−0.74	0.12914
6002	8.1064	112.786	342.417	0.02257	0.001	−0.77	0.11441
6003	8.1065	112.954	341.979	0.02252	0.001	−0.89	0.10058
6004	8.1066	113.118	341.557	0.02247	0.001	−1.08	0.08763
6005	8.1067	113.267	341.170	0.02253	0.001	−0.72	0.07556
6006	8.1068	113.403	340.814	0.02246	0.001	−0.95	0.06439
6018	7.5842	105.344	342.791	0.02238	0.001	−0.76	0.12412
6019	7.5843	105.453	342.487	0.02233	0.001	−0.94	0.11439
6020	7.5845	105.566	342.175	0.02236	0.001	−0.76	0.10507
6021	7.5846	105.664	341.892	0.02236	0.001	−0.70	0.09614
6022	7.5845	105.762	341.615	0.02228	0.001	−0.98	0.08761
6023	7.5848	105.859	341.349	0.02232	0.001	−0.76	0.07948
6024	7.5848	105.949	341.101	0.02231	0.001	−0.79	0.07173
6036	6.9060	95.828	342.896	0.02210	0.001	−0.89	0.12412
6037	6.9061	95.933	342.567	0.02208	0.001	−0.89	0.11440
6038	6.9062	96.027	342.270	0.02199	0.002	−1.27	0.10508
6039	6.9065	96.128	341.971	0.02205	0.001	−0.95	0.09615
6040	6.9062	96.214	341.683	0.02212	0.001	−0.55	0.08761
6041	6.9064	96.304	341.419	0.02200	0.001	−1.05	0.07948
6042	6.9065	96.386	341.167	0.02198	0.002	−1.07	0.07173
6054	6.2153	86.152	342.983	0.02187	0.001	−0.77	0.12412
6055	6.2153	86.241	342.663	0.02188	0.001	−0.66	0.11440
6056	6.2154	86.327	342.362	0.02186	0.001	−0.66	0.10508
6057	6.2155	86.413	342.061	0.02173	0.002	−1.20	0.09615
6058	6.2156	86.499	341.768	0.02184	0.001	−0.67	0.08762
6059	6.2155	86.573	341.498	0.02182	0.001	−0.68	0.07948
6060	6.2156	86.651	341.226	0.02185	0.001	−0.49	0.07174
6072	5.5214	76.433	343.103	0.02158	0.001	−0.93	0.12412
6073	5.5215	76.518	342.765	0.02155	0.001	−1.01	0.11440
6074	5.5216	76.597	342.443	0.02158	0.001	−0.82	0.10507
6075	5.5215	76.675	342.137	0.02153	0.001	−0.95	0.09615
6076	5.5216	76.749	341.833	0.02153	0.001	−0.92	0.08761
6077	5.5215	76.819	341.552	0.02151	0.001	−0.96	0.07948
6078	5.5216	76.886	341.288	0.02155	0.001	−0.72	0.07173
6090	4.8340	66.823	343.216	0.02135	0.001	−0.90	0.12412
6091	4.8341	66.901	342.864	0.02134	0.001	−0.85	0.11440
6092	4.8341	66.971	342.531	0.02128	0.001	−1.06	0.10508
6093	4.8340	67.038	342.223	0.02124	0.001	−1.19	0.09614
6094	4.8341	67.104	341.915	0.02132	0.001	−0.76	0.08762
6095	4.8342	67.170	341.622	0.02125	0.001	−1.02	0.07948
6096	4.8341	67.225	341.353	0.02121	0.001	−1.14	0.07174
6108	4.1410	57.158	343.345	0.02106	0.001	−1.12	0.12414
6109	4.1411	57.221	342.987	0.02107	0.001	−0.98	0.11441
6110	4.1409	57.280	342.651	0.02106	0.001	−0.98	0.10508
6112	4.1408	57.397	342.005	0.02100	0.001	−1.12	0.08762
6113	4.1410	57.455	341.706	0.02095	0.001	−1.31	0.07949
6114	4.1411	57.506	341.425	0.02105	0.001	−0.77	0.07174
6126	3.4501	47.568	343.297	0.02085	0.001	−0.97	0.11921
6127	3.4501	47.619	342.946	0.02077	0.001	−1.30	0.10969
6128	3.4499	47.670	342.599	0.02071	0.001	−1.49	0.10057
6130	3.4495	47.760	341.955	0.02080	0.001	−0.94	0.08351
6131	3.4496	47.806	341.653	0.02077	0.001	−0.99	0.07556
6132	3.4496	47.849	341.371	0.02074	0.001	−1.10	0.06802
6144	3.4481	47.537	343.306	0.02081	0.001	−1.18	0.11923
6145	3.4481	47.592	342.944	0.02078	0.001	−1.25	0.10970
6146	3.4483	47.646	342.606	0.02075	0.001	−1.30	0.10058
6147	3.4481	47.693	342.273	0.02072	0.001	−1.37	0.09184
6148	3.4481	47.740	341.956	0.02078	0.001	−1.03	0.08351
6149	3.4481	47.783	341.663	0.02074	0.001	−1.18	0.07556
6150	3.4481	47.826	341.373	0.02074	0.001	−1.08	0.06802
6162	2.7654	38.056	343.459	0.02057	0.001	−1.27	0.11920
6163	2.7655	38.103	343.089	0.02057	0.001	−1.17	0.10968
6164	2.7655	38.142	342.738	0.02049	0.001	−1.52	0.10056
6165	2.7656	38.189	342.377	0.02049	0.001	−1.41	0.09184
6166	2.7655	38.224	342.061	0.02055	0.001	−1.03	0.08350
6167	2.7655	38.259	341.746	0.02050	0.001	−1.25	0.07557
6168	2.7655	38.294	341.445	0.02050	0.001	−1.17	0.06802
6191	2.0713	28.462	343.464	0.02017	0.001	−2.15	0.11439
6192	2.0713	28.497	343.073	0.02025	0.001	−1.65	0.10508
6193	2.0713	28.528	342.708	0.02021	0.001	−1.79	0.09615
6194	2.0714	28.560	342.363	0.02021	0.001	−1.67	0.08762
6195	2.0712	28.587	342.028	0.02024	0.001	−1.43	0.07948
6196	2.0712	28.614	341.716	0.02016	0.001	−1.78	0.07174
6197	2.0713	28.642	341.421	0.02022	0.001	−1.40	0.06439
6198	1.3694	18.786	343.481	0.01995	0.001	−2.15	0.10968
6199	1.3694	18.809	343.095	0.01993	0.001	−2.17	0.10055
6200	1.3694	18.829	342.723	0.01988	0.001	−2.29	0.09182
6201	1.3694	18.848	342.368	0.01989	0.001	−2.19	0.08350
6202	1.3694	18.868	342.026	0.01992	0.001	−1.93	0.07555
6203	1.3695	18.888	341.699	0.01988	0.001	−2.09	0.06801
6204	1.3694	18.903	341.405	0.01976	0.002	−2.63	0.06086
6216	0.6979	9.559	343.548	0.01966	0.001	−2.61	0.10506
6217	0.6979	9.567	343.146	0.01963	0.001	−2.64	0.09613
6218	0.6978	9.578	342.756	0.01958	0.001	−2.84	0.08761
6219	0.6978	9.590	342.387	0.01959	0.001	−2.66	0.07947
6220	0.6979	9.602	342.037	0.01960	0.001	−2.53	0.07173
6221	0.6978	9.610	341.703	0.01952	0.002	−2.89	0.06439
6222	0.6978	9.618	341.387	0.01955	0.002	−2.64	0.05744
6234	0.3486	4.764	343.883	0.01963	0.002	−2.27	0.10505
6235	0.3485	4.772	343.440	0.01963	0.001	−2.18	0.09612
6236	0.3486	4.776	343.023	0.01966	0.002	−1.94	0.08760
6237	0.3485	4.779	342.632	0.01959	0.001	−2.18	0.07947
6238	0.3485	4.787	342.259	0.01965	0.002	−1.80	0.07173
6239	0.3485	4.791	341.896	0.01959	0.002	−2.02	0.06438
6240	0.3486	4.799	341.572	0.01968	0.003	−1.46	0.05743
6252	0.1686	2.304	343.987	0.01986	0.002	−0.86	0.10052
6253	0.1686	2.304	343.531	0.01986	0.002	−0.76	0.09180
6254	0.1686	2.308	343.096	0.01984	0.002	−0.75	0.08346
6255	0.1685	2.312	342.690	0.01982	0.002	−0.73	0.07553
6256	0.1685	2.312	342.308	0.01986	0.002	−0.44	0.06800
6257	0.1685	2.316	341.932	0.01983	0.002	−0.51	0.06085
6258	0.1685	2.319	341.589	0.01984	0.003	−0.36	0.05410

**Table 3:** Thermal conductivity of argon, steady-state method

Point no.	*p* (MPa)	*ρ* (kg m^−3^)	*T*_exp_ (K)	λ_exp_ (W m^−1^ K^−1^)	*TBAND*	Relative dev. (%)	*q* (W m^−1^)	Δ*T*	*Ra*
Nominal temperature 300 K									

3015	7.4443	123.423	300.647	0.02051	1.33	−0.89	0.01420	0.663	51646
3016	7.4439	123.407	300.667	0.02082	1.45	0.58	0.01582	0.722	56238
3017	7.4442	123.391	300.713	0.02071	1.42	0.05	0.01753	0.796	61990
3018	7.4439	123.363	300.757	0.02030	1.10	−1.97	0.01933	0.886	68872
3034	6.9070	114.295	300.621	0.02093	1.46	2.31	0.01267	0.591	39372
3035	6.9071	114.283	300.651	0.02070	1.44	1.20	0.01420	0.665	44239
3036	6.9069	114.263	300.694	0.02010	1.61	−1.79	0.01582	0.755	50224
3038	6.9073	114.235	300.766	0.02042	1.12	−0.17	0.01933	0.894	59373
3039	6.9074	114.215	300.812	0.02034	1.81	−0.58	0.02122	0.977	64788
3051	6.2656	103.318	300.866	0.02016	1.21	−0.12	0.02319	1.088	58667
3052	6.2652	103.326	300.830	0.01989	1.43	−1.44	0.02122	1.015	54769
3053	6.2653	103.345	300.788	0.01994	1.13	−1.20	0.01933	0.930	50217
3054	6.2652	103.357	300.747	0.01993	1.22	−1.26	0.01754	0.850	45946
3055	6.2654	103.377	300.709	0.01989	1.63	−1.45	0.01583	0.774	41870
3056	6.2654	103.393	300.672	0.02019	1.53	0.08	0.01420	0.690	37329
3058	6.2654	103.425	300.592	0.02079	1.77	2.97	0.01122	0.537	29104
3071	5.5847	91.824	300.867	0.02017	1.18	1.37	0.02319	1.109	46858
3072	5.5847	91.836	300.831	0.02023	1.13	1.69	0.02122	1.018	43048
3073	5.5848	91.856	300.783	0.02025	1.27	1.81	0.01933	0.933	39457
3074	5.5848	91.872	300.738	0.02031	1.07	2.10	0.01753	0.849	35936
3075	5.5848	91.884	300.700	0.02029	1.57	2.01	0.01582	0.771	32670
3076	5.5847	91.896	300.663	0.02032	1.46	2.17	0.01420	0.695	29460
3077	5.5846	91.908	300.625	0.02036	1.50	2.36	0.01267	0.622	26388
3094	4.9524	81.270	300.629	0.02002	1.65	1.96	0.01267	0.639	21009
3095	4.9525	81.258	300.670	0.01997	1.85	1.71	0.01421	0.715	23490
3096	4.9525	81.246	300.709	0.01995	1.32	1.63	0.01583	0.793	26058
3097	4.9526	81.234	300.751	0.01997	1.56	1.71	0.01754	0.874	28690
3098	4.9526	81.222	300.797	0.01990	1.49	1.38	0.01933	0.962	31549
3099	4.9527	81.210	300.845	0.01995	1.12	1.59	0.02122	1.048	34349
3100	4.9528	81.198	300.887	0.01990	1.38	1.36	0.02319	1.142	37404
3113	4.2565	69.625	300.610	0.01891	1.77	−2.28	0.01122	0.606	14468
3115	4.2560	69.601	300.674	0.01944	1.26	0.46	0.01420	0.742	17689
3116	4.2561	69.589	300.727	0.01938	1.18	0.18	0.01583	0.826	19675
3117	4.2561	69.581	300.764	0.01945	1.13	0.49	0.01754	0.909	21640
3118	4.2562	69.569	300.812	0.01947	1.35	0.58	0.01933	0.997	23725
3119	4.2562	69.561	300.846	0.01951	1.20	0.79	0.02122	1.088	25868
3120	4.2561	69.545	300.902	0.01950	1.37	0.72	0.02319	1.185	28147
3134	3.5546	57.937	300.649	0.01889	1.41	−0.98	0.01267	0.689	11239
3135	3.5547	57.929	300.680	0.01925	1.44	0.89	0.01420	0.756	12333
3136	3.5546	57.917	300.725	0.01925	1.78	0.89	0.01583	0.841	13697
3137	3.5545	57.909	300.770	0.01924	0.95	0.82	0.01754	0.929	15132
3138	3.5544	57.897	300.814	0.01923	0.90	0.75	0.01933	1.023	16635
3139	3.5544	57.885	300.865	0.01923	1.10	0.71	0.02122	1.119	18194
3140	3.5544	57.877	300.910	0.01924	1.23	0.76	0.02319	1.219	19801
4013	2.8395	46.096	300.725	0.01865	1.54	−0.89	0.01122	0.623	6343
4014	2.8395	46.092	300.758	0.01865	1.52	−0.90	0.01267	0.703	7145
4015	2.8395	46.084	300.807	0.01863	1.22	−1.01	0.01420	0.787	7999
4016	2.8395	46.076	300.844	0.01870	1.48	−0.68	0.01582	0.873	8863
4017	2.8395	46.068	300.897	0.01871	0.89	−0.62	0.01753	0.965	9788
4018	2.8394	46.060	300.938	0.01874	0.91	−0.46	0.01933	1.060	10745
4019	2.8392	46.048	300.987	0.01875	0.95	−0.45	0.02121	1.161	11757
4020	2.8392	46.040	301.037	0.01879	1.12	−0.23	0.02319	1.264	12786
4033	2.1134	34.180	300.707	0.01864	1.92	0.45	0.01123	0.627	3453
4034	2.1132	34.172	300.753	0.01841	1.89	−0.82	0.01267	0.716	3941
4035	2.1133	34.168	300.795	0.01863	1.40	0.35	0.01421	0.793	4360
4036	2.1132	34.160	300.840	0.01861	1.24	0.23	0.01583	0.884	4855
4037	2.1132	34.152	300.885	0.01858	1.48	0.04	0.01754	0.980	5379
4038	2.1132	34.148	300.932	0.01851	0.95	−0.34	0.01934	1.084	5942
4039	2.1132	34.140	300.990	0.01843	0.85	−0.78	0.02123	1.193	6535
4040	2.1132	34.132	301.050	0.01833	0.99	−1.32	0.02320	1.309	7164
4053	1.3696	22.059	300.691	0.01830	1.67	−0.04	0.01123	0.641	1445
4054	1.3697	22.055	300.739	0.01789	1.76	−2.34	0.01267	0.740	1667
4055	1.3696	22.051	300.789	0.01791	1.31	−2.24	0.01421	0.829	1864
4056	1.3698	22.051	300.822	0.01826	1.17	−0.29	0.01583	0.905	2036
4057	1.3696	22.047	300.869	0.01830	0.82	−0.09	0.01754	1.001	2248
4058	1.3698	22.043	300.922	0.01826	0.86	−0.30	0.01934	1.105	2481
4059	1.3698	22.043	300.975	0.01825	0.75	−0.38	0.02122	1.213	2721
4060	1.3698	22.035	301.030	0.01825	1.04	−0.39	0.02320	1.325	2970
4061	1.3700	22.035	301.091	0.01826	0.52	−0.38	0.02526	1.442	3229
4062	1.3698	22.027	301.147	0.01822	0.66	−0.60	0.02741	1.567	3504
4063	1.3698	22.023	301.211	0.01821	0.59	−0.70	0.02964	1.695	3788
4064	1.3699	22.019	301.275	0.01822	0.61	−0.62	0.03197	1.825	4075
4065	1.3699	22.015	301.353	0.01810	0.67	−1.32	0.03438	1.975	4404
4066	1.3699	22.007	301.417	0.01811	0.57	−1.29	0.03688	2.116	4714
4067	1.3700	22.003	301.494	0.01812	0.45	−1.27	0.03947	2.262	5034
4068	1.3701	21.999	301.569	0.01811	0.50	−1.31	0.04214	2.415	5367
4069	1.3699	21.991	301.641	0.01818	0.46	−0.94	0.04491	2.562	5686
4070	1.3700	21.987	301.724	0.01817	0.46	−1.01	0.04776	2.724	6039
4072	0.7011	11.249	300.661	0.01834	1.79	1.37	0.01123	0.641	369
4073	0.7010	11.245	300.753	0.01794	1.36	−0.85	0.01421	0.829	477
4075	0.7012	11.241	300.957	0.01799	1.24	−0.65	0.02122	1.235	708
4076	0.7010	11.229	301.197	0.01802	0.59	−0.55	0.02964	1.722	983
4077	0.7009	11.221	301.325	0.01811	0.47	−0.07	0.03438	1.986	1131
4078	0.7010	11.217	301.470	0.01810	0.53	−0.18	0.03947	2.281	1297
4079	0.7011	11.213	301.632	0.01807	0.39	−0.34	0.04491	2.597	1473
4080	0.7011	11.205	301.799	0.01807	0.34	−0.41	0.05070	2.932	1658
4093	0.3781	6.056	300.717	0.01820	1.87	1.14	0.01421	0.818	135
4094	0.3780	6.052	300.816	0.01815	1.06	0.85	0.01754	1.013	167
4095	0.3780	6.048	300.919	0.01810	0.84	0.53	0.02122	1.229	202
4096	0.3780	6.044	301.043	0.01807	0.72	0.38	0.02526	1.464	241
4097	0.3780	6.044	301.170	0.01806	0.53	0.26	0.02964	1.720	282
4098	0.3780	6.040	301.307	0.01802	0.39	0.03	0.03438	1.998	327
4099	0.3780	6.040	301.447	0.01802	0.32	−0.04	0.03947	2.294	375
4100	0.3781	6.036	301.614	0.01801	0.28	−0.13	0.04491	2.612	426
4111	0.1684	2.692	300.811	0.01765	0.95	−1.56	0.01754	1.041	34
4112	0.1685	2.692	300.920	0.01771	0.79	−1.28	0.02122	1.256	41
4113	0.1686	2.692	301.025	0.01790	0.68	−0.24	0.02526	1.479	48
4114	0.1686	2.692	301.159	0.01789	0.56	−0.34	0.02964	1.737	56
4115	0.1685	2.689	301.295	0.01789	0.42	−0.36	0.03438	2.014	65
4116	0.1684	2.689	301.447	0.01787	0.32	−0.49	0.03946	2.314	74
4117	0.1685	2.689	301.601	0.01787	0.36	−0.53	0.04490	2.633	85
4118	0.1684	2.685	301.772	0.01787	0.22	−0.61	0.05070	2.974	95
4119	0.1685	2.685	301.958	0.01787	0.16	−0.66	0.05684	3.333	106
4120	0.1686	2.685	302.138	0.01787	0.20	−0.69	0.06333	3.713	118

Nominal temperature 320 K									

5011	8.3286	128.181	320.414	0.02219	1.42	1.21	0.01519	0.662	45411
5012	8.3287	128.165	320.448	0.02212	1.07	0.89	0.01693	0.734	50310
5013	8.3287	128.149	320.483	0.02212	1.37	0.88	0.01876	0.807	55252
5014	8.3288	128.133	320.524	0.02198	1.06	0.24	0.02068	0.887	60707
5015	8.3287	128.109	320.566	0.02184	1.02	−0.40	0.02270	0.970	66368
5031	7.5812	116.492	320.447	0.02190	1.34	1.34	0.01693	0.752	42454
5032	7.5812	116.480	320.478	0.02187	1.06	1.19	0.01876	0.828	46742
5033	7.5813	116.460	320.521	0.02181	0.79	0.91	0.02068	0.908	51240
5034	7.5815	116.444	320.564	0.02175	1.32	0.64	0.02270	0.992	55906
5035	7.5815	116.428	320.604	0.02165	0.92	0.18	0.02480	1.080	60833
5036	7.5816	116.412	320.644	0.02159	0.85	−0.11	0.02701	1.169	65840
5051	6.8718	105.431	320.434	0.02165	1.12	1.57	0.01693	0.769	35481
5052	6.8721	105.419	320.474	0.02164	1.04	1.53	0.01876	0.847	39058
5053	6.8719	105.403	320.510	0.02161	0.90	1.39	0.02068	0.930	42813
5054	6.8720	105.383	320.564	0.02154	1.28	1.03	0.02269	1.017	46793
5055	6.8722	105.367	320.607	0.02148	0.94	0.77	0.02480	1.106	50882
5056	6.8722	105.355	320.644	0.02141	0.83	0.41	0.02701	1.200	55164
5059	6.8724	105.299	320.793	0.02125	0.98	−0.34	0.03418	1.497	68604
5071	6.1864	94.785	320.337	0.02154	1.90	2.37	0.01355	0.632	23485
5072	6.1863	94.769	320.377	0.02145	1.25	1.96	0.01519	0.708	26285
5073	6.1864	94.757	320.422	0.02144	1.29	1.91	0.01693	0.785	29134
5074	6.1863	94.745	320.459	0.02141	1.24	1.76	0.01876	0.867	32135
5075	6.1864	94.725	320.510	0.02097	1.17	−0.32	0.02068	0.970	35906
5076	6.1863	94.709	320.556	0.02105	1.14	0.09	0.02269	1.054	39002
5077	6.1863	94.697	320.596	0.02103	0.85	−0.04	0.02480	1.147	42405
5078	6.1861	94.677	320.645	0.02103	0.88	−0.03	0.02701	1.241	45850
5079	6.1860	94.661	320.689	0.02102	1.00	−0.09	0.02930	1.339	49437
5080	6.1860	94.641	320.741	0.02099	0.78	−0.28	0.03169	1.442	53179
5091	5.5066	84.234	320.284	0.02101	1.96	1.21	0.01200	0.581	16950
5092	5.5067	84.222	320.324	0.02080	1.92	0.24	0.01355	0.659	19231
5093	5.5068	84.210	320.360	0.02105	1.38	1.40	0.01519	0.728	21218
5094	5.5065	84.194	320.403	0.02101	1.09	1.19	0.01693	0.809	23571
5095	5.5066	84.186	320.437	0.02102	1.41	1.22	0.01875	0.893	25981
5096	5.5068	84.174	320.483	0.02102	1.14	1.22	0.02068	0.980	28495
5097	5.5067	84.158	320.530	0.02099	0.97	1.05	0.02269	1.072	31151
5098	5.5070	84.150	320.573	0.02087	1.06	0.51	0.02480	1.172	34039
5099	5.5071	84.138	320.627	0.02073	1.05	−0.18	0.02700	1.278	37084
5100	5.5070	84.119	320.682	0.02075	1.03	−0.08	0.02930	1.378	39960
5112	4.8436	73.944	320.298	0.02081	1.50	1.48	0.01355	0.665	14844
5113	4.8438	73.940	320.334	0.02078	1.77	1.37	0.01519	0.743	16599
5114	4.8438	73.932	320.369	0.02077	1.07	1.28	0.01693	0.826	18436
5115	4.8438	73.916	320.420	0.02077	1.08	1.27	0.01876	0.912	20342
5116	4.8439	73.908	320.463	0.02077	0.98	1.26	0.02068	1.002	22330
5117	4.8438	73.892	320.506	0.02080	1.17	1.39	0.02269	1.095	24367
5118	4.8440	73.884	320.554	0.02088	1.13	1.79	0.02480	1.187	26408
5119	4.8441	73.876	320.597	0.02088	1.44	1.78	0.02700	1.287	28623
5120	4.8442	73.864	320.650	0.02094	1.17	2.05	0.02930	1.388	30823
5133	4.1375	63.018	320.337	0.02050	1.40	1.27	0.01519	0.760	12223
5134	4.1375	63.010	320.384	0.02044	1.31	1.01	0.01693	0.847	13608
5135	4.1375	62.998	320.437	0.02039	1.13	0.75	0.01876	0.938	15063
5136	4.1377	62.994	320.471	0.02045	1.20	1.02	0.02068	1.029	16509
5137	4.1377	62.982	320.521	0.02041	0.92	0.83	0.02269	1.128	18085
5138	4.1376	62.970	320.572	0.02041	1.54	0.83	0.02480	1.229	19691
5139	4.1375	62.954	320.633	0.02044	1.12	0.95	0.02700	1.332	21327
5140	4.1373	62.942	320.679	0.02046	1.39	1.01	0.02930	1.440	23035
5152	3.4547	52.508	320.300	0.02025	1.74	1.30	0.01355	0.692	7658
5153	3.4547	52.500	320.342	0.02021	1.41	1.11	0.01519	0.776	8580
5154	3.4548	52.496	320.386	0.02017	0.99	0.91	0.01693	0.864	9555
5155	3.4549	52.488	320.434	0.02009	0.95	0.49	0.01875	0.960	10602
5156	3.4551	52.484	320.479	0.02012	1.11	0.61	0.02068	1.055	11644
5157	3.4549	52.468	320.537	0.02001	1.07	0.07	0.02269	1.161	12807
5158	3.4551	52.464	320.584	0.02004	1.47	0.23	0.02480	1.264	13936
5159	3.4549	52.452	320.641	0.02009	0.75	0.43	0.02700	1.371	15094
5160	3.4552	52.444	320.702	0.01995	1.06	−0.27	0.02930	1.494	16438
5171	2.7635	41.893	320.349	0.01968	1.43	−0.32	0.01519	0.801	5582
5172	2.7635	41.889	320.396	0.01965	1.00	−0.50	0.01693	0.893	6219
5173	2.7635	41.881	320.444	0.01961	1.07	−0.71	0.01875	0.990	6891
5174	2.7635	41.873	320.491	0.01961	1.04	−0.73	0.02067	1.090	7581
5175	2.7635	41.866	320.552	0.01957	0.80	−0.95	0.02269	1.197	8319
5176	2.7635	41.862	320.597	0.01955	0.78	−1.05	0.02480	1.308	9081
5177	2.7637	41.854	320.653	0.01960	0.80	−0.80	0.02700	1.418	9841
5178	2.7638	41.846	320.719	0.01961	1.14	−0.76	0.02930	1.536	10646
5179	2.7636	41.838	320.775	0.01964	1.30	−0.64	0.03169	1.657	11471
5180	2.7637	41.830	320.833	0.01966	1.15	−0.55	0.03418	1.782	12328
5182	2.0779	31.419	320.394	0.01944	1.20	−0.38	0.01693	0.907	3514
5183	2.0781	31.415	320.444	0.01937	1.26	−0.73	0.01876	1.008	3901
5184	2.0781	31.411	320.495	0.01937	0.92	−0.75	0.02068	1.110	4295
5185	2.0781	31.403	320.549	0.01935	0.81	−0.86	0.02269	1.219	4710
5186	2.0782	31.399	320.606	0.01933	0.86	−0.99	0.02480	1.332	5146
5187	2.0782	31.395	320.663	0.01933	0.55	−1.01	0.02700	1.450	5594
5188	2.0783	31.391	320.729	0.01930	0.64	−1.19	0.02930	1.574	6070
5189	2.0783	31.383	320.785	0.01929	0.58	−1.24	0.03169	1.702	6555
5190	2.0783	31.375	320.850	0.01929	0.64	−1.28	0.03417	1.834	7057
5203	1.3798	20.813	320.327	0.01947	1.39	1.00	0.01519	0.816	1370
5204	1.3798	20.805	320.434	0.01934	1.14	0.29	0.01875	1.013	1699
5205	1.3796	20.797	320.538	0.01926	0.81	−0.14	0.02269	1.230	2059
5207	1.3798	20.781	320.791	0.01913	0.83	−0.86	0.03169	1.727	2880
5208	1.3798	20.773	320.925	0.01909	0.69	−1.14	0.03675	2.006	3338
5209	1.3800	20.765	321.070	0.01915	0.47	−0.85	0.04218	2.293	3809
5210	1.3798	20.753	321.229	0.01912	0.44	−1.03	0.04799	2.610	4324
5222	0.6847	10.303	320.238	0.01937	1.94	1.67	0.01200	0.649	264
5223	0.6849	10.303	320.324	0.01933	1.23	1.42	0.01519	0.823	334
5224	0.6849	10.299	320.418	0.01927	1.26	1.09	0.01875	1.019	413
5225	0.6848	10.295	320.528	0.01912	0.78	0.30	0.02269	1.243	503
5226	0.6849	10.291	320.656	0.01900	1.01	−0.39	0.02700	1.488	601
5227	0.6847	10.283	320.784	0.01898	0.80	−0.52	0.03169	1.748	704
5228	0.6849	10.283	320.930	0.01897	0.42	−0.58	0.03675	2.027	816
5229	0.6848	10.275	321.074	0.01900	0.69	−0.49	0.04218	2.324	932
5230	0.6849	10.275	321.232	0.01901	0.48	−0.45	0.04799	2.641	1058
5244	0.3681	5.529	320.427	0.01875	1.06	−1.09	0.01875	1.048	122
5245	0.3680	5.525	320.532	0.01880	1.25	−0.90	0.02269	1.265	146
5246	0.3680	5.525	320.645	0.01892	1.29	−0.26	0.02700	1.495	173
5247	0.3680	5.521	320.776	0.01891	0.79	−0.33	0.03169	1.755	203
5248	0.3680	5.517	320.911	0.01889	0.81	−0.48	0.03675	2.038	235
5249	0.3680	5.517	321.067	0.01888	0.60	−0.56	0.04218	2.340	269
5250	0.3680	5.513	321.230	0.01888	0.74	−0.61	0.04799	2.662	305
5261	0.3679	5.505	321.489	0.01888	0.67	−0.67	0.05741	3.185	363
5262	0.3680	5.501	321.774	0.01880	0.58	−1.21	0.06767	3.770	429
5263	0.3680	5.497	322.089	0.01882	0.65	−1.19	0.07877	4.384	496
5264	0.3680	5.493	322.413	0.01883	0.60	−1.23	0.09071	5.045	568
5265	0.1734	2.601	320.774	0.01872	0.65	−1.06	0.03169	1.774	45
5266	0.1733	2.597	320.913	0.01870	0.31	−1.20	0.03675	2.060	52
5267	0.1734	2.597	321.063	0.01871	0.28	−1.20	0.04218	2.363	60
5268	0.1734	2.597	321.227	0.01872	0.29	−1.17	0.04799	2.687	68
5269	0.1733	2.593	321.396	0.01877	0.21	−0.92	0.05418	3.024	76
5270	0.1732	2.589	321.582	0.01879	0.13	−0.91	0.06073	3.388	85
5271	0.1733	2.589	321.769	0.01879	0.19	−0.95	0.06767	3.775	95
5272	0.1733	2.589	321.972	0.01878	0.13	−1.03	0.07497	4.184	105
5273	0.1732	2.585	322.196	0.01879	0.11	−1.03	0.08265	4.609	115
5274	01733	2.585	322.410	0.01879	0.17	−1.11	0.09071	5.060	126

Nominal temperature 340 K									

6008	8.1066	116.696	339.086	0.02319	1.78	2.52	0.01524	0.652	31009
6009	8.1065	116.660	339.178	0.02313	0.99	2.27	0.01991	0.840	39919
6010	8.1065	116.616	339.287	0.02289	0.95	1.20	0.02519	1.056	50097
6011	8.1063	116.564	339.407	0.02266	0.71	0.19	0.03110	1.293	61186
6026	7.5849	109.126	339.099	0.02281	1.87	1.82	0.01524	0.666	27701
6027	7.5850	109.094	339.192	0.02273	1.12	1.45	0.01991	0.861	35747
6028	7.5849	109.050	339.305	0.02260	0.82	0.88	0.02519	1.079	44718
6029	7.5848	109.006	339.427	0.02247	0.86	0.28	0.03110	1.318	54503
6030	7.5849	108.958	339.556	0.02229	0.54	−0.55	0.03762	1.579	65176
6044	6.9067	99.287	339.107	0.02271	1.83	2.53	0.01524	0.675	23177
6045	6.9067	99.255	339.209	0.02258	1.21	1.95	0.01991	0.876	30031
6046	6.9070	99.223	339.317	0.02255	1.04	1.83	0.02519	1.095	37496
6047	6.9070	99.179	339.450	0.02241	0.75	1.16	0.03110	1.342	45834
6048	6.9070	99.135	339.582	0.02228	0.70	0.57	0.03763	1.608	54818
6049	6.9067	99.083	339.726	0.02210	0.80	−0.28	0.04477	1.898	64562
6062	6.2157	89.268	339.122	0.02160	1.92	−1.22	0.01524	0.714	19754
6063	6.2156	89.232	339.235	0.02179	1.31	−0.39	0.01991	0.915	25283
6064	6.2156	89.204	339.340	0.02220	0.82	1.44	0.02519	1.125	31042
6065	6.2153	89.164	339.462	0.02208	0.89	0.89	0.03110	1.380	37987
6066	6.2155	89.120	339.610	0.02187	0.83	−0.08	0.03762	1.663	45688
6067	6.2156	89.080	339.753	0.02183	0.90	−0.30	0.04477	1.956	53623
6068	6.2156	89.032	339.914	0.02173	0.71	−0.77	0.05254	2.273	62158
6080	5.5216	79.213	339.130	0.02173	1.57	0.59	0.01524	0.715	15518
6081	5.5217	79.185	339.241	0.02171	0.86	0.48	0.01991	0.927	20083
6082	5.5218	79.157	339.358	0.02175	1.11	0.60	0.02519	1.161	25109
6083	5.5219	79.125	339.487	0.02177	0.95	0.67	0.03110	1.418	30609
6084	5.5218	79.089	339.618	0.02175	0.83	0.54	0.03763	1.699	36611
6085	5.5219	79.049	339.774	0.02173	0.99	0.43	0.04477	2.001	43020
6086	5.5219	79.009	339.942	0.02166	0.68	0.09	0.05254	2.328	49920
6087	5.5218	78.965	340.106	0.02164	1.03	−0.04	0.06092	2.670	57107
6088	5.5219	78.913	340.301	0.02154	0.82	−0.54	0.06993	3.040	64822
6098	4.8343	69.270	339.143	0.02138	1.49	0.14	0.01524	0.731	12075
6099	4.8342	69.246	339.248	0.02142	1.13	0.27	0.01991	0.947	15621
6100	4.8341	69.214	339.376	0.02143	0.92	0.31	0.02519	1.189	19576
6101	4.8341	69.186	339.505	0.02152	0.86	0.67	0.03110	1.451	23842
6102	4.8341	69.154	339.648	0.02155	1.14	0.78	0.03762	1.739	28507
6103	4.8341	69.118	339.810	0.02156	1.33	0.80	0.04477	2.050	33527
6104	4.8340	69.082	339.973	0.02154	1.68	0.68	0.05254	2.386	38917
6116	4.1414	59.263	339.161	0.02069	1.87	−2.02	0.01525	0.760	9130
6117	4.1415	59.243	339.262	0.02103	1.17	−0.37	0.01991	0.971	11658
6118	4.1415	59.219	339.391	0.02109	0.97	−0.14	0.02520	1.220	14608
6119	4.1416	59.195	339.521	0.02113	0.71	0.05	0.03110	1.494	17856
6120	4.1417	59.167	339.668	0.02124	1.23	0.54	0.03763	1.786	21312
6121	4.1415	59.135	339.834	0.02131	1.51	0.81	0.04478	2.105	25051
6133	3.4497	49.312	339.056	0.02065	1.89	−1.00	0.01120	0.564	4664
6134	3.4499	49.296	339.158	0.02069	1.49	−0.82	0.01525	0.763	6306
6135	3.4499	49.280	339.273	0.02069	0.93	−0.87	0.01991	0.994	8195
6136	3.4498	49.256	339.405	0.02070	0.90	−0.85	0.02519	1.252	10305
6137	3.4499	49.236	339.544	0.02079	1.02	−0.42	0.03110	1.532	12586
6138	3.4499	49.212	339.704	0.02082	1.09	−0.32	0.03763	1.843	15102
6139	3.4501	49.192	339.859	0.02091	1.43	0.06	0.04477	2.173	17771
6140	3.4499	49.160	340.041	0.02097	1.57	0.29	0.05255	2.531	20635
6152	3.4481	49.272	339.157	0.02064	1.18	−1.09	0.01525	0.765	6316
6153	3.4481	49.252	339.278	0.02066	1.16	−1.00	0.01991	0.995	8196
6154	3.4481	49.232	339.408	0.02068	0.64	−0.92	0.02520	1.253	10302
6155	3.4481	49.212	339.550	0.02079	0.76	−0.42	0.03110	1.532	12572
6156	3.4483	49.188	339.706	0.02081	0.86	−0.40	0.03763	1.844	15100
6157	3.4483	49.164	339.869	0.02090	1.32	0.02	0.04478	2.175	17759
6158	3.4483	49.136	340.042	0.02102	2.00	0.53	0.05254	2.525	20568
6170	2.7656	39.461	339.156	0.02078	1.30	0.73	0.01525	0.763	4010
6171	2.7655	39.445	339.283	0.02044	1.12	−0.97	0.01991	1.011	5301
6172	2.7657	39.429	339.424	0.02044	0.73	−0.98	0.02519	1.276	6677
6173	2.7658	39.413	339.566	0.02045	0.68	−0.96	0.03110	1.570	8199
6174	2.7658	39.393	339.727	0.02048	0.75	−0.86	0.03763	1.891	9854
6175	2.7657	39.369	339.889	0.02059	1.30	−0.37	0.04477	2.232	11599
6176	2.7658	39.349	340.086	0.02062	1.14	−0.26	0.05254	2.606	13507
6177	2.7658	39.325	340.282	0.02070	1.70	0.07	0.06093	3.000	15503
6178	2.7659	39.301	340.493	0.02076	1.60	0.34	0.06993	3.419	17619
6180	2.0708	29.506	339.062	0.02022	1.90	−0.88	0.01120	0.579	1687
6181	2.0708	29.498	339.165	0.02020	1.40	−0.97	0.01524	0.788	2292
6182	2.0710	29.490	339.293	0.02015	1.08	−1.26	0.01991	1.030	2991
6183	2.0710	29.478	339.423	0.02018	0.63	−1.16	0.02519	1.300	3768
6184	2.0711	29.462	339.580	0.02017	0.51	−1.24	0.03110	1.602	4636
6185	2.0710	29.446	339.741	0.02018	0.58	−1.24	0.03763	1.935	5584
6186	2.0710	29.434	339.922	0.02016	0.52	−1.38	0.04477	2.300	6622
6187	2.0710	29.414	340.117	0.02018	0.53	−1.29	0.05254	2.690	7722
6188	2.0711	29.398	340.325	0.02026	0.92	−0.97	0.06092	3.101	8878
6189	2.0712	29.378	340.536	0.02036	1.41	−0.53	0.06993	3.535	10089
6190	2.0712	29.358	340.775	0.02037	1.04	−0.51	0.07955	4.009	11403
6206	1.3695	19.475	339.154	0.02012	1.49	−0.24	0.01525	0.793	996
6207	1.3695	19.467	339.277	0.02011	1.01	−0.32	0.01991	1.035	1298
6208	1.3696	19.459	339.418	0.02007	0.72	−0.55	0.02519	1.312	1641
6209	1.3696	19.451	339.566	0.02007	0.69	−0.63	0.03110	1.619	2022
6210	1.3696	19.443	339.742	0.02005	0.63	−0.75	0.03763	1.958	2440
6211	1.3696	19.431	339.926	0.02003	0.61	−0.89	0.04477	2.330	2896
6212	1.3696	19.419	340.130	0.02001	0.47	−1.07	0.05254	2.736	3390
6213	1.3696	19.407	340.348	0.02000	0.46	−1.16	0.06092	3.171	3917
6214	1.3696	19.391	340.575	0.01999	0.43	−1.26	0.06993	3.637	4479
6215	1.3696	19.379	340.835	0.01997	0.39	−1.39	0.07955	4.136	5074
6224	0.6978	9.903	339.155	0.01963	1.44	−1.67	0.01525	0.814	262
6225	0.6980	9.903	339.273	0.01996	1.01	−0.04	0.01991	1.045	336
6226	0.6979	9.899	339.413	0.01995	0.71	−0.12	0.02519	1.323	424
6227	0.6980	9.895	339.567	0.01993	0.74	−0.24	0.03110	1.634	523
6228	0.6979	9.887	339.741	0.01992	0.54	−0.33	0.03763	1.977	631
6229	0.6977	9.883	339.920	0.01992	0.55	−0.41	0.04477	2.353	749
6230	0.6977	9.875	340.129	0.01987	0.49	−0.69	0.05254	2.767	878
6231	0.6977	9.867	340.354	0.01986	0.50	−0.80	0.06092	3.209	1015
6232	0.6977	9.859	340.586	0.01990	0.50	−0.64	0.06993	3.675	1158
6233	0.6977	9.855	340.836	0.01989	0.46	−0.76	0.07955	4.182	1314
6242	0.3486	4.942	339.149	0.01970	1.77	−0.79	0.01525	0.811	65
6243	0.3485	4.942	339.275	0.01969	0.99	−0.86	0.01991	1.060	84
6244	0.3487	4.942	339.406	0.01969	0.88	−0.89	0.02519	1.341	107
6245	0.3486	4.938	339.576	0.01969	0.54	−0.92	0.03110	1.655	131
6246	0.3487	4.938	339.744	0.01970	0.64	−0.91	0.03763	2.001	158
6247	0.3487	4.934	339.933	0.01970	0.56	−0.98	0.04477	2.382	188
6248	0.3487	4.930	340.138	0.01969	0.52	−1.04	0.05254	2.795	220
6249	0.3487	4.926	340.359	0.01972	0.43	−0.97	0.06092	3.237	254
6250	0.3486	4.922	340.601	0.01974	0.45	−0.92	0.06992	3.711	290
6251	0.3487	4.922	340.854	0.01974	0.38	−0.99	0.07954	4.221	329
6260	0.1685	2.389	339.169	0.01924	1.78	−2.89	0.01525	0.830	15
6261	0.1685	2.389	339.292	0.01943	1.08	−1.94	0.01991	1.074	20
6262	0.1685	2.385	339.430	0.01954	0.64	−1.41	0.02519	1.352	25
6263	0.1685	2.385	339.589	0.01960	0.56	−1.11	0.03110	1.663	31
6264	0.1685	2.385	339.760	0.01964	0.40	−0.94	0.03763	2.008	37
6265	0.1684	2.381	339.948	0.01966	0.33	−0.90	0.04477	2.387	44
6266	0.1684	2.381	340.158	0.01966	0.25	−0.96	0.05254	2.801	51
6267	0.1684	2.377	340.381	0.01967	0.23	−0.94	0.06092	3.246	59
6268	0.1685	2.377	340.621	0.01968	0.16	−0.98	0.06992	3.724	68
6269	0.1684	2.377	340.870	0.01969	0.15	−0.98	0.07955	4.235	77

**Table 4:** Steady-state results with single wires

Point no.	*p* (MPa)	*ρ* (kg m^−3^)	*T*_exp_ (K)	λ_exp_ (W m^−1^ K^−1^)	*TBAND*	Relative Dev. (%)	*q* (W m^−1^)	*Ra*
Long hot wire, high pressure								

8001	2.0753	29.546	339.323	0.02038	1.60	−0.14	0.01991	2970
8001	2.0753	29.554	339.255	0.02352	0.00	13.25	0.01989	2575
8002	2.0752	29.542	339.382	0.02034	1.37	−0.36	0.02195	3276
8002	2.0752	29.550	339.304	0.02362	0.00	13.60	0.02193	2824
8003	2.0754	29.538	339.440	0.02033	1.17	−0.43	0.02409	3593
8003	2.0754	29.546	339.367	0.02305	0.00	11.43	0.02407	3172
8004	2.0754	29.534	339.491	0.02031	1.28	−0.52	0.02633	3925
8004	2.0754	29.542	339.408	0.02316	0.00	11.88	0.02631	3445
8005	2.0752	29.526	339.557	0.02031	0.99	−0.56	0.02867	4267
8005	2.0752	29.534	339.479	0.02269	0.00	10.02	0.02864	3822
8006	2.0754	29.522	339.612	0.02025	0.90	−0.84	0.03111	4636
8006	2.0754	29.530	339.533	0.02248	0.00	9.16	0.03108	4181
8007	2.0754	29.514	339.683	0.02023	0.87	−0.96	0.03364	5010
8007	2.0754	29.522	339.604	0.02225	0.00	8.22	0.03361	4560
8008	2.0754	29.510	339.748	0.02022	0.68	−1.02	0.03628	5396
8008	2.0754	29.518	339.666	0.02216	0.00	7.85	0.03624	4929
8009	2.0755	29.506	339.819	0.02024	0.76	−0.93	0.03901	5788
8009	2.0755	29.514	339.733	0.02214	0.00	7.72	0.03898	5299
8010	2.0755	29.498	339.898	0.02021	0.75	−1.12	0.04185	6207
8010	2.0755	29.506	339.817	0.02186	0.00	6.55	0.04181	5743
10001	2.0757	29.494	339.967	0.02023	0.59	−1.06	0.04478	6627
10001	2.0757	29.502	339.888	0.02171	0.00	5.87	0.04474	6180
10002	2.0759	29.490	340.038	0.02021	0.55	−1.17	0.04781	7070
10002	2.0759	29.498	339.964	0.02150	0.00	4.92	0.04776	6651
10003	2.0759	29.482	340.120	0.02022	0.72	−1.11	0.05095	7513
10003	2.0759	29.490	340.034	0.02164	0.00	5.52	0.05090	7030
10004	2.0759	29.478	340.197	0.02022	0.73	−1.14	0.05417	7974
10004	2.0759	29.482	340.122	0.02138	0.00	4.38	0.05412	7547
10005	2.0759	29.470	340.283	0.02026	0.99	−0.97	0.05750	8431
10005	2.0759	29.474	340.211	0.02131	0.00	4.02	0.05745	8023
10006	2.0760	29.462	340.360	0.02028	1.01	−0.88	0.06093	8908
10006	2.0760	29.470	340.290	0.02123	0.01	3.65	0.06087	8516
10007	2.0759	29.454	340.455	0.02033	1.57	−0.64	0.06446	9379
10007	2.0759	29.462	340.380	0.02130	0.01	3.95	0.06440	8959
10008	2.0759	29.446	340.539	0.02036	1.47	−0.54	0.06809	9873
10008	2.0759	29.450	340.465	0.02125	0.01	3.73	0.06802	9463
10009	2.0761	29.438	340.630	0.02037	1.38	−0.51	0.07181	10386
10009	2.0761	29.446	340.556	0.02123	0.01	3.58	0.07174	9974
10010	2.0761	29.430	340.724	0.02038	1.37	−0.46	0.07564	10907
10010	2.0761	29.438	340.639	0.02133	0.01	4.04	0.07557	10432

Long hot wire, low pressure								

8001	0.1697	2.405	339.253	0.01988	1.31	0.37	0.01991	20
8001	0.1697	2.405	339.160	0.02417	0.00	18.06	0.01989	16
8002	0.1697	2.405	339.305	0.01985	1.37	0.23	0.02195	22
8002	0.1697	2.405	339.225	0.02298	0.00	13.82	0.02193	19
8003	0.1697	2.405	339.359	0.01985	1.11	0.18	0.02409	24
8003	0.1697	2.405	339.279	0.02267	0.00	12.65	0.02406	21
8004	0.1697	2.405	339.425	0.01984	0.99	0.12	0.02633	26
8004	0.1697	2.405	339.345	0.02241	0.00	11.59	0.02630	23
8005	0.1697	2.405	339.477	0.01983	0.88	0.09	0.02866	28
8005	0.1697	2.405	339.410	0.02173	0.00	8.85	0.02863	26
8006	0.1697	2.405	339.544	0.01981	0.80	−0.05	0.03110	31
8006	0.1697	2.405	339.470	0.02173	0.00	8.83	0.03107	28
8007	0.1698	2.405	339.611	0.01982	0.67	−0.03	0.03364	33
8007	0.1698	2.405	339.540	0.02153	0.00	7.96	0.03360	31
8008	0.1696	2.401	339.682	0.01980	0.69	−0.15	0.03627	36
8008	0.1696	2.401	339.603	0.02155	0.00	8.01	0.03624	33
8009	0.1697	2.401	339.748	0.01980	0.65	−0.14	0.03901	39
8009	0.1697	2.401	339.678	0.02124	0.00	6.64	0.03897	36
8010	0.1696	2.401	339.827	0.01979	0.48	−0.23	0.04184	41
8010	0.1696	2.401	339.757	0.02111	0.00	6.07	0.04180	39

Short hot wire, high pressure								

9001	2.0756	29.550	339.322	0.02048	1.73	0.36	0.01991	2956
9001	2.0756	29.562	339.220	0.02577	0.00	20.81	0.01999	2365
9002	2.0757	29.550	339.379	0.02041	1.36	−0.01	0.02195	3267
9002	2.0757	29.558	339.267	0.02567	0.00	20.51	0.02204	2615
9003	2.0757	29.542	339.433	0.02039	1.55	−0.12	0.02409	3583
9003	2.0757	29.554	339.318	0.02521	0.00	19.03	0.02419	2919
9004	2.0758	29.538	339.487	0.02038	1.15	−0.20	0.02633	3914
9004	2.0758	29.550	339.371	0.02475	0.00	17.52	0.02644	3245
9005	2.0758	29.534	339.548	0.02033	1.12	−0.44	0.02867	4265
9005	2.0758	29.546	339.420	0.02478	0.00	17.63	0.02878	3524
9006	2.0757	29.526	339.613	0.02031	1.10	−0.57	0.03110	4625
9006	2.0757	29.538	339.474	0.02476	0.00	17.54	0.03123	3822
9007	2.0757	29.522	339.680	0.02029	0.94	−0.66	0.03364	4997
9007	2.0757	29.534	339.542	0.02432	0.00	16.04	0.03377	4200
9008	2.0758	29.518	339.746	0.02026	0.89	−0.85	0.03628	5389
9008	2.0758	29.530	339.583	0.02471	0.00	17.37	0.03642	4453
9009	2.0757	29.510	339.817	0.02023	0.78	−0.98	0.03901	5792
9009	2.0757	29.526	339.644	0.02461	0.00	17.01	0.03917	4802
9010	2.0758	29.502	339.884	0.02022	0.78	−1.04	0.04185	6205
9010	2.0758	29.522	339.697	0.02466	0.00	17.15	0.04202	5135
11001	2.0765	29.506	339.950	0.02019	0.58	−1.22	0.04478	6644
11001	2.0765	29.526	339.759	0.02437	0.00	16.18	0.04496	5552
11002	2.0766	29.502	340.028	0.02019	0.83	−1.25	0.04781	7081
11002	2.0766	29.522	339.805	0.02486	0.00	17.80	0.04801	5807
11003	2.0767	29.498	340.106	0.02020	0.76	−1.20	0.05094	7527
11003	2.0767	29.518	339.868	0.02490	0.00	17.94	0.05115	6167
11004	2.0766	29.490	340.184	0.02021	0.73	−1.21	0.05417	7987
11004	2.0766	29.510	339.940	0.02470	0.00	17.24	0.05439	6601
11005	2.0766	29.482	340.266	0.02023	0.94	−1.10	0.05750	8450
11005	2.0766	29.502	340.011	0.02464	0.00	17.06	0.05774	7009
11006	2.0766	29.474	340.357	0.02026	1.04	−0.96	0.06093	8921
11006	2.0766	29.498	340.091	0.02464	0.00	17.02	0.06118	7415
11007	2.0766	29.466	340.440	0.02031	1.60	−0.77	0.06446	9398
11007	2.0766	29.490	340.163	0.02462	0.00	16.94	0.06472	7837
11008	2.0768	29.458	340.523	0.02033	1.45	−0.66	0.06808	9894
11008	2.0768	29.486	340.243	0.02445	0.00	16.33	0.06836	8320
11009	2.0768	29.450	340.622	0.02035	1.52	−0.57	0.07181	10401
11009	2.0768	29.478	340.320	0.02461	0.00	16.87	0.07211	8703
11010	2.0768	29.442	340.706	0.02035	1.39	−0.59	0.07563	10930
11010	2.0768	29.470	340.392	0.02455	0.00	16.65	0.07595	9171

Voltmeter across the bridge								

7001	0.1695	2.401	339.238	0.01977	1.19	−0.18	0.01991	20
7001	0.1695	2.401	339.238	0.01977	0.20	−0.18	0.01991	20
7002	0.1696	2.405	339.289	0.01979	0.96	−0.07	0.02195	22
7002	0.1696	2.405	339.289	0.01979	0.15	−0.07	0.02195	22
7003	0.1697	2.405	339.346	0.01978	0.77	−0.14	0.02409	24
7003	0.1697	2.405	339.346	0.01978	0.14	−0.14	0.02409	24
7004	0.1697	2.405	339.401	0.01978	1.07	−0.16	0.02632	26
7004	0.1697	2.405	339.401	0.01978	0.15	−0.16	0.02632	26
7005	0.1698	2.405	339.460	0.01978	0.89	−0.18	0.02866	28
7005	0.1698	2.405	339.460	0.01978	0.13	−0.18	0.02866	28
7006	0.1697	2.405	339.531	0.01976	0.57	−0.28	0.03110	31
7006	0.1697	2.405	339.531	0.01976	0.12	−0.28	0.03110	31
7007	0.1697	2.401	339.597	0.01975	0.54	−0.36	0.03363	33
7007	0.1697	2.401	339.597	0.01975	0.06	−0.36	0.03363	33
7008	0.1697	2.401	339.661	0.01976	0.57	−0.34	0.03627	36
7008	0.1697	2.401	339.661	0.01976	0.11	−0.34	0.03627	36
7009	0.1697	2.401	339.733	0.01977	0.42	−0.27	0.03900	39
7009	0.1697	2.401	339.733	0.01977	0.11	−0.27	0.03900	39
7010	0.1697	2.401	339.815	0.01975	0.41	−0.42	0.04184	41
7010	0.1697	2.401	339.815	0.01975	0.13	−0.42	0.04184	41

Vacuum, short hot wire, very low pressure								

12001	0.0013	0.020	338.819	0.00472	25.82	−318.00	0.00125	0
12001	0.0013	0.020	338.901	0.00297	0.14	−564.60	0.00125	0
12002	0.0013	0.020	339.281	0.00431	2.59	−358.20	0.00498	0
12002	0.0013	0.020	338.941	0.00995	0.07	−98.29	0.00500	0
12003	0.0014	0.020	340.050	0.00426	0.90	−364.60	0.01119	0
12003	0.0014	0.020	339.964	0.00456	0.04	−334.00	0.01124	0
12004	0.0015	0.020	341.128	0.00423	0.56	−369.00	0.01989	0
12004	0.0015	0.020	340.812	0.00488	0.03	−306.40	0.01997	0
12005	0.0014	0.020	342.520	0.00422	0.38	−371.80	0.03105	0
12005	0.0014	0.020	342.110	0.00474	0.03	−319.60	0.03118	0

**Table 5:** Averaged thermal conductivity of argon, adjusted to even temperature

Averaged transient data			Averaged steady-state data		
*ρ* (kg m^−3^)	λ_exp_ (W m^−1^ K^−1^)	Dev. from line (%)	*ρ* (kg m^−3^)	λ_exp_ (W m^−1^ K^−1^)	Dev. from line (%)
Nominal temperature 300 K					

122.4886	0.02067	0.09	123.395	0.02055	−0.60
113.6361	0.02043	−0.01	114.259	0.02046	0.06
102.7463	0.02015	−0.05	103.361	0.02007	−0.53
91.3571	0.01984	−0.19	91.868	0.02024	1.73
80.7629	0.01956	−0.28	81.234	0.01991	1.42
69.1979	0.01922	−0.56	69.581	0.01934	0.02
57.5930	0.01892	−0.63	57.909	0.01915	0.54
45.8204	0.01868	−0.35	46.072	0.01865	−0.55
33.9558	0.01831	−0.77	34.156	0.01847	0.07
21.9155	0.01800	−0.85	22.027	0.01811	−0.25
11.1655	0.01778	−0.59	11.229	0.01802	0.74
6.0162	0.01752	−1.35	6.044	0.01802	1.45
2.6765	0.01781	0.76	2.689	0.01777	0.54

Nominal temperature 320 K					

127.462	0.02181	−0.48	128.149	0.02202	0.40
115.574	0.02146	−0.65	116.452	0.02173	0.50
104.588	0.02120	−0.51	105.379	0.02148	0.71
93.974	0.02087	−0.75	94.717	0.02116	0.54
83.579	0.02057	−0.88	84.178	0.02090	0.64
73.472	0.02035	−0.65	73.908	0.02080	1.47
62.646	0.02008	−0.57	62.982	0.02041	1.01
52.152	0.01982	−0.49	52.480	0.02008	0.77
41.654	0.01955	−0.45	41.862	0.01959	−0.28
31.367	0.01920	−0.86	31.399	0.01931	−0.29
20.677	0.01889	−1.02	20.785	0.01918	0.49
10.227	0.01867	−0.73	10.291	0.01908	1.43
5.481	0.01851	−0.92	5.513	0.01879	0.58
2.573	0.01868	0.41	2.593	0.01868	0.41

Nominal temperature 340 K					

115.629	0.02243	−0.85	116.632	0.02301	1.57
108.091	0.02223	−0.82	109.046	0.02262	0.80
98.332	0.02194	−0.92	99.195	0.02247	1.35
88.401	0.02173	−0.64	89.156	0.02190	0.05
78.434	0.02144	−0.71	79.077	0.02171	0.46
68.579	0.02117	−0.71	69.182	0.02151	0.80
58.660	0.02091	−0.65	59.203	0.02111	0.23
48.800	0.02065	−0.60	49.228	0.02081	0.11
39.061	0.02040	−0.52	39.389	0.02059	0.37
29.214	0.02009	−0.72	29.442	0.02023	−0.05
19.283	0.01977	−0.96	19.431	0.02005	0.43
9.811	0.01947	−1.17	9.883	0.01989	0.96
4.890	0.01951	−0.27	4.934	0.01971	0.74
2.365	0.01971	1.10	2.381	0.01958	0.45

**Table 6:** Experimental and theoretical dilute-gas thermal conductivity and first-density coefficients^a^ for argon. Here, *U* is the expanded uncertainty (coverage factor *k* = 2, and thus a 2 standard deviation estimate)

*T* (K)	λ_0_ (exp) (W m^−1^ K^−1^)	2*U* (W m^−1^ K^−1^)	λ_0_^b^ (W m^−1^ K^−1^)	Relative dev. (%)	λ_0_^c^ (W m^−1^ K^−1^)	Relative dev. (%)	λ_0_^d^ (W m^−1^ K^−1^)	Relative dev. (%)
Dilute-gas thermal conductivity								

300	0.01761	0.00010	0.01761	0	0.01784	−1.30	0.01772	−0.62
320	0.01853	0.00010	0.01871	−0.92	0.01883	−1.62	0.01870	−0.92
340	0.01943	0.00010			0.01979	−1.85	0.01966	−1.18

*T* (K)	λ_1_ (exp) (W m^−1^ K^−1^)	2*U* (W m^−1^ K^−1^)	λ_1_^b^ (W m^−1^ K^−1^)	Relative dev. (%)	λ_1_^e^ (W m^−1^ K^−1^)	Relative dev. (%)	λ_1_^f^ (W m^−1^ K^−1^)	Relative dev. (%)

First-density coefficient								

300	0.00099	0.00006	0.00106	−7.07	0.00105	−5.74	0.00110	−10.77
320	0.00106	0.00006	0.00106	0	0.00104	1.79	0.00110	3.87
340	0.00110	0.00007			0.00104	5.71	0.00110	0.27

a λ_1_ = (*∂λ*/*∂ρ*)_T_ W L mol^−1^ m^−1^ K^−1^

b Ref. [22]

c Ref. [14]

d Ref. [15]

e Refs. [16,17]

f Ref. [18]

**Table 7:** Survey of the valid temperature rises

*T* (K)	Transient		Steady state	
	No. points	Δ*T*_mean_ (K)	No. points	Δ*T*_mean_ (K)
300	98	2.082	104	1.261
320	105	2.215	120	1.473
340	102	3.378	119	1.826
